# One-step flow synthesis and [^68^Ga]Ga radiolabelling of metal phenolic network nanoparticles for *in vivo* PET tracking

**DOI:** 10.1039/d6nr01151a

**Published:** 2026-08-27

**Authors:** Esmé Shepherd, Amaia Carrascal Miniño, Aishwarya Mishra, Juan Pellico, Jason Sengel, Rafael T. M. de Rosales, Andrew J. Surman

**Affiliations:** a Research Department of Imaging Chemistry and Biology, School of Biomedical Engineering & Imaging Sciences, King's College London 4th Floor Lambeth Wing St Thomas’ Hospital London SE1 7EH UK rafael.torres@kcl.ac.uk; b Department of Chemistry, Faculty of Natural, Mathematical & Engineering Sciences, King's College London Britannia House 7 Trinity Street London SE1 1DB UK andrew.surman@kcl.ac.uk

## Abstract

Metal-phenolic network (MPN) nanoparticles are growing in popularity as drug delivery vehicles due to their versatile binding interactions, with increased attention to their applications for positron emission tomography (PET) due to their inherent ability to coordinate radiometals. Whilst the ability to do this has only been shown with long-lived radionuclides such as ^89^Zr and ^64^Cu, the use of short-lived radionuclides remain unexplored due to the lengthy syntheses required. Fast incorporation of a radionuclide into a flow synthesis could alleviate this hurdle, producing a one-step, facile synthesis for PET imaging of these nanomaterials. Here we report the synthesis of MPN-based nanoparticles (67 ± 1 nm) using a flow mixing apparatus called a multi-inlet vortex mixer (MIVM). The particle structure consists of a [^68^Ga]Ga-doped Fe(iii)–tannic acid (TA) metal–phenolic network (MPN) core encapsulating the drug molecule rapamycin, with a polymer shell consisting of hyaluronic acid which has been modified with dopamine (HAd) (FRITH-MPNPs). A post-synthesis radiolabelling approach was implemented using [^68^Ga]Ga^3+^ with a high radiochemical yield (>68%), purity (>96%) and human serum stability (>97%), proving the ability to use these systems as PET imaging agents. To incorporate the radiolabel more stably within the core, a novel method is presented, directly incorporating the radiometal into the flow synthesis, creating a PET imaging guided drug delivery agent in a one-step process. [^68^Ga]Ga-FRITH-MPNPs radiolabelled *via* flow have an increased radiochemical yield (84%) compared with [^68^Ga]Ga-FRITH-MPNPs radiolabelled *via* the post-synthesis radiolabelling approach (68%), as this provides access to binding sites in the core of the particle as well as decorating the surface. Furthermore, [^68^Ga]Ga-FRITH-MPNPs radiolabelled *via* flow maintain high radiochemical purity (97%) and serum stability (97%). These radiolabelled particles were characterised and compared to non-labelled FRITH-MPNPs using DLS, TEM, encapsulation efficiency *via* HPLC, ICP-MS, PXRD and zeta potential, which all show that radiolabelling does not significantly impact the structure. *In vitro* cytotoxicity of [^68^Ga]Ga-FRITH-MPNPs on J774a.1 macrophages was negligible. PET imaging in a healthy mice showed high stability *in vivo*. Overall, this work shows that MPN-based nanoparticles called [^68^Ga]Ga-FRITH-MPNPs can be effectively synthesised and simultaneously radiolabelled using a flow method, and imaged using PET/CT for future applications in immune system targeting.

## Abstract 2

Metal-phenolic network nanoparticles are growing in popularity as drug delivery vehicles. One-step flow synthesis facilitates the incorporation of short-lived radionuclides, for PET imaging of these nanomaterials.

## Graphical abstract

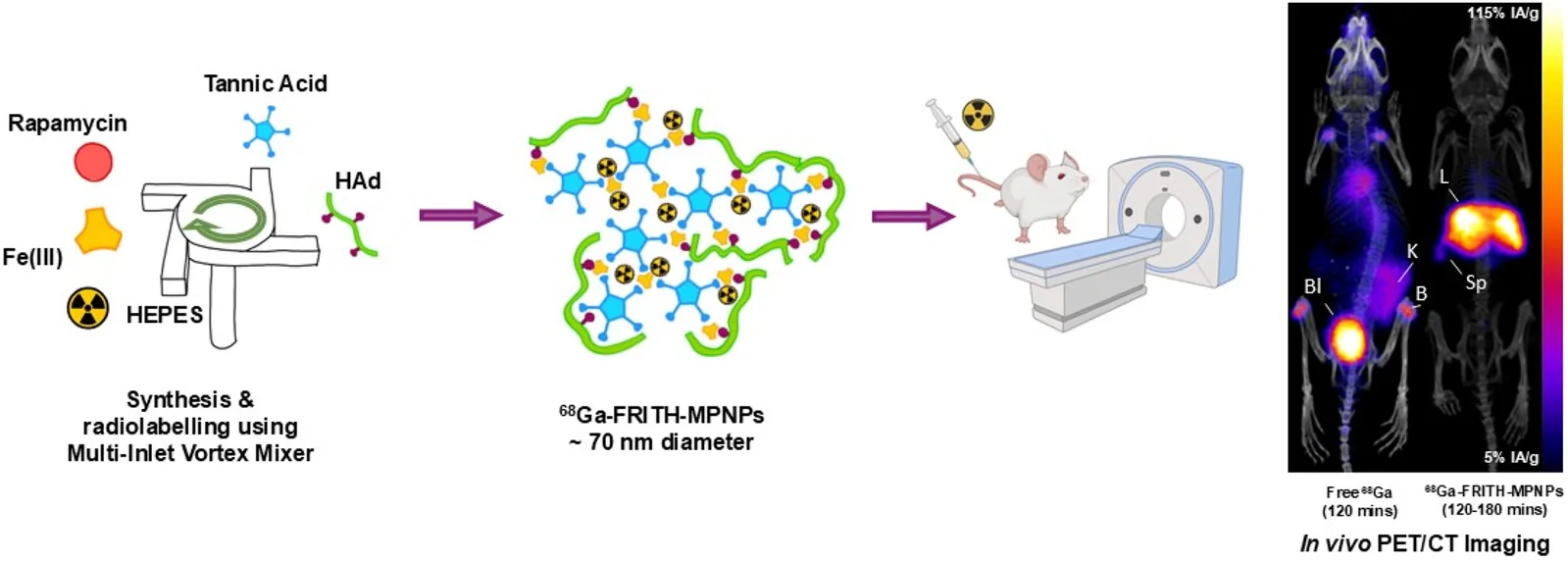

## Introduction

Nanoparticles have emerged as a versatile tool in theranostics – the integrated approach combining therapeutic and diagnostic capabilities within a single platform. By combining this with targeted drug delivery, the efficacy of treatment can be enhanced whilst decreasing undesired side effects, and providing real-time *in vivo* monitoring of particle accumulation in relevant tissues through imaging modalities.^1,2^

Compared with the nanoparticle systems currently available for theranostics, metal phenolic networks (MPN) have several properties that make them suitable for development.^3^ MPNs are supramolecular, crosslinked networks formed through the coordination of metal ions and phenolic ligands. They can incorporate hydrophobic drug molecules into their structure, provided there are sufficient functional groups with the means to coordinate metals. Combined with their high biocompatibility and ability to undergo functionalisation, MPNs are promising candidates for targeted therapies.

Caruso *et al.* first reported MPNs as thin film materials in 2013, and later transformed them into nanoparticles by depositing them onto a substrate, which was subsequently dissolved, resulting in a hollow nanoparticle structure.^4^ Following this, Liu *et al.* reported that nanoparticles can be formed with an MPN core rather than as a hollow shell, due to the supramolecular nature of MPNs, meaning they can be self-assembled into nanoparticles *via* non-covalent interactions.^4–6^ They were assembled through a flash nanoprecipitation (FNP) process using a multi-inlet vortex mixer (MIVM) with four inlets and programmable syringe pumps,^7,8^ enabling large scale and rapid production whilst maintaining control over the size, shape and composition of the nanoparticles. They showed that these particles could be used both as MRI contrast agents thanks to the ferric ions in the MPN core, and for fluorescence imaging by harnessing particle autofluorescence when doxorubicin was incorporated. This showed that the blood circulation time of doxorubicin was increased by incorporating it into the MPN particle, as well as enhanced tumour accumulation.

Several medical imaging techniques allow the imaging of nanoparticles *in vivo*,^9^ and whilst magnetic resonance imaging (MRI) is more commonly used for non-invasive diagnostics, positron emission tomography (PET) is rapidly emerging, due to its high sensitivity, low mass requirement for radiotracer, accurate quantification and the ability to image on the whole-body scale. To achieve PET imaging, the particle or molecule that is being tracked needs to be labelled with a radionuclide to be detected by PET cameras.

Techniques have been developed to radiolabel inorganic particles for PET imaging, typically involving a post-synthesis incubation of the particles with radiometals, which coordinate to functional groups on the surface.^10^ However, this can adversely affect the targeting and drug release of the particle if the isotope alters the surface chemistry, which may lead to a change in particle behaviour.^11^ Additionally, there are few approaches that incorporate the radionuclide into the particle during synthesis.^12–16^ Development of a simultaneous synthesis and radiolabelling would provide a facile, rapid and scalable manufacturing approach that can readily be used in a clinical setting.

There are several reports using PET to image MPNs, all using either ^64^Cu or ^89^Zr, and radiolabelling by incubating the particles with the radionuclide post-synthesis, or bulk mixing the particle components with the radionuclide.^14–19^ Meanwhile, MPN nanoparticles or capsules have been produced by a flow method using microfluidics, which utilises laminar flow,^20,21^ and *via* the MIVM apparatus based on turbulent mixing.^6^ Flow synthesis enables more control over the addition of each component and produces more uniform particles and size control through adjusting flow rates. We hypothesised that these methods could be combined by self-assembling an MPN-based nanoparticle in a flow mixer alongside a PET isotope in a one-step synthesis, creating highly tuneable and uniform particles for nuclear imaging. Furthermore, flow synthesis would enable the use of short-lived isotopes, which would reduce radiation exposure.

**Fig. 1:**
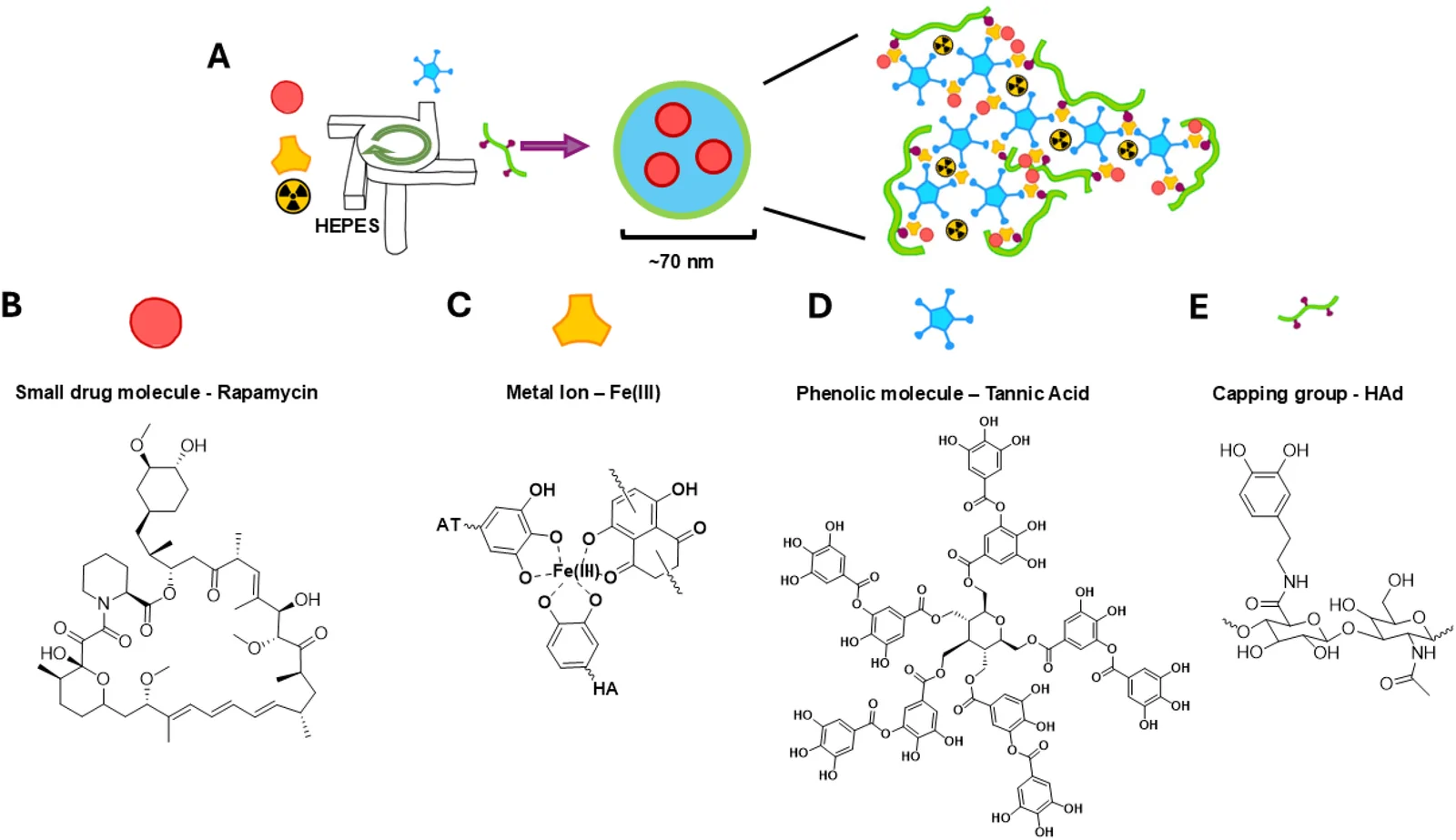
(A) Schematic showing synthesis and theoretical structure of [^68^Ga]Ga-FRITH-MPNPs from the individual components using a flow mixer; (B) chemical structure of the drug molecule rapamycin; (C) octahedral structure of the iron coordination centre with other particle components formed around it; (D) chemical structure of the phenolic molecule tannic acid (TA); (E) chemical structure of the capping group hyaluronic acid modified with dopamine (HAd).

Here we report the development and characterisation of [^68^Ga]Ga-FRITH-MPNPs (Fig. 1A) ([^68^Ga]Ga-labelled flow-synthesised rapamycin (Fig. 1B), iron(iii) (Fig. 1C), tannic acid (TA) (Fig. 1D), HAd (Fig. 1E), metal–phenolic nanoparticles) with a Fe(iii)–TA core, doped with short-lived [^68^Ga]Ga(iii) for PET imaging. The small drug molecule rapamycin is encapsulated, and the surface is functionalised for targeting using hyaluronic acid modified with dopamine (HAd) to enhance its metal binding properties. The particles were synthesised using a syringe pump-controlled multi-inlet vortex mixer (MIVM) with four ports allowing precise control over the addition of each component, providing radiolabelled nanoparticles in a single step. The particles were tracked *in vivo* using PET imaging in a healthy murine model to assess whether the radiolabelling method provided radiochemical stability and biocompatibility appropriate for future use as a drug delivery agent.

## Materials & methods

### Materials

#### Multi-inlet vortex mixer (MIVM) and flow mixing set-up

The mixer was formed of two plates which were designed in Computer Aided Design (CAD) software (SolidWorks 2006 SP4.1) and broadly based on dimensions used previously published designs.^22–26^ The main mixing channel has 1 mm width channels from the wells to the central mixing space with a 5 mm diameter and ∼1 mm hole for the resulting particles to be eluted through. The well size and thickness of the structures are not widely reported in previously published designs, and were optimised according here to the resilience of the material and forces from the desired flow rates. The plates were cut from 10 mm clear cast acrylic PMMA (Sheet Plastics), using a CNC milling machine (Roland Moddela MDX-40). Inlet and outlet ports had screw threads cut into them using a tapping tool (1/4″ × 28 UNF HSSGT STR. Flute plug tap, Sherwood, manufacturer's part number (MPN SHF-085-1110C)) to enable connection of adapters directly to the mixer.

The plates were secured together using M6 × 30 mm hex socket cap screw stainless steel (MPN: 290146), M6 steel hex nuts (MPN: 2010852) and M6 stainless steel washers (MPN: 189658) (RS pro); ethylene tetrafluoroethylene (ETFE) tubing (0.093″, IDEX, MPN: 02017-39) was connected between the syringe pumps and mixer using female luer to female adapters (ETFE, luer to ¼-28 flat bottom 0.040″ bore, IDEX, MPN: 02014-03), flangeless male nuts (PEEK, ¼″-28 Flat-bottom 1/8″ OD, IDEX, MPN 02020-16), and flangeless ferrules (1/8″, Kinesis, MPN: 02019-82).

Digitally controlled syringe pumps (Harvard Apparatus, PHD 2000 programmable) were used to control the injection of particle components into the mixer, and two luer lock syringes (Luer lock 50 mL, diameter 29.100 nm, Terumo) were installed to each pump to connect to the adapters.

Photographs of the mixer plates, set-up and full CAD designs can be found in the SI.

#### FRITH-MPNPs synthesis

30–50 kDa MWCO Hyaluronic acid (HA) (BioServUK Limited); HCl, HEPES buffer, Iron(iii) chloride hexahydrate (FeCl_3_·6H_2_O), Tannic acid (TA), Tween 20, HPLC grade methanol, WST-8 (Merck Life science UK Limited); 1-ethyl-3-(3-dimethylaminopropyl)carbodiimide (EDC), *N*-hydroxysuccinimide (NHS), dopamine hydrochloride, rapamycin, D_2_O (Fluorochem); 10 kDa SnakeSkin dialysis tubing (ThermoScientific); formic acid (Fisher Scientific UK Ltd); 30 kDa Amicon Ultra 0.5 centrifugal filter units (MilliporeSigma); sartorius 0.2 μm syringe filters with cellulose acetate membranes.

#### Radiolabelling

Gallium-68 was eluted as [^68^Ga]GaCl_3_ from two Eckert & Ziegler [^68^Ge]/[^68^Ga] generators; ultrapure HCl (5 mL, 0.1 M) manufactured to good manufacturing practice (GMP) requirements (ABX, Germany); manual SEC purification was performed using a PD MiniTrap™ G-25 Medium size exclusion column containing 2.1 mL Sephadex resin (Global Life Sciences Solutions Operations UK Ltd).

#### *In vivo* and *in vitro* studies

Phosphate buffered saline (PBS), Dulbecco's modified Eagle's High Glucose medium (DMEM), fetal bovine serum (FBS), 1 mM sodium pyruvate, 2 mM l-glutamine, penicillin–streptomycin, were all obtained from ThermoFisher Scientific; mouse monocyte macrophages J774a.1 (Rayne Institute, Cryostore); BALB/c mice, female, 8–10 weeks, 20 g, (Charles River UK); human serum (human male AB plasma, USA origin, sterile-filtered, Sigma-Aldrich, USA).

#### Equipment

The UV/Vis spectrometer used was an Agilent Cary 100 UV-Vis spectrometer (200 nm–400 nm, lamp change 350 nm, double beam) and the NMR spectrometer used was a Bruker Ascend 400; lyophilisation of HAd was performed using a Labconco Free Zone 4.5 L −85 °C benchtop freeze drier. Radioactivity for all samples was measured in a gamma counter (LKB Wallac 1282 Compugamma CS) using EdenTerm software or dose calibrator (Capintec. Inc.). Hydrodynamic size and zeta potential were measured on a Zetasizer NanoZS90 (Malvern Instruments) at 25 °C. The Centre for Ultrastructural Imaging (CUI) was used to visualise the particles using a JEOL-1400 Plus transmission electron microscope (TEM) at a voltage of 120 kV. The centrifuge used was an Eppendorf centrifuge 5418. The metal content measurements were conducted on a PerkinElmer NexION 5000 Inductively Coupled Plasma Triple Quadrupole Mass Spectrometer (ICP-QQQMS) under Dynamic Reaction Cell (DRC) mode at the London Metallomics Facility, King's College London. The introduction system to the instrument was a Cetac ASX-560 autosampler coupled to a SeaSpray glass nebulizer fitted to a quartz cyclonic spray chamber. Argon plasma flow and nebulizer gas flow rates were 18 L min^−1^ and 0.98 L min^−1^, respectively. Powder diffraction data was collected using a Bruker D8 Venture Eco powder diffractometer with Cu Kα radiation (*λ* = 1.5406 Å) operating at 40 kV and 25 mA, using *θ*/2*θ* geometry, with 2*θ* range from 5 to 50°, and speed of 0.25° s^−1^ and steps of 0.2°. All samples were mounted using a zero-background sample holder, where the powders (freeze dried unpurified FRITH-MPNPs, and reactants) were mounted in the centre of the holder, while the FRITH-MPNPs were drop casted from a solution with the buffer over the sample holder and left for drying before data collection.

Rapamycin content was measured using an Agilent 1260 Infinity II HPLC, and a reverse phase C-18 column (Agilent Poroshell 120 EC-C18, pore size 2.7 μm, internal diameter 4.6 mm, length 50 mm). The UV/Vis of *in vitro* cell assay plates was measured using a BMG Labtech Spectrostar Nano spectrometer at 450 nm; PET/CT images were acquired using a NanoPET/CT scanner (Mediso) with 4-mouse hotel bed; images were reconstructed using Tera-Tomo 3D reconstruction (400–600 keV energy window, 1 : 5 coincidence mode, 20 iterations and 1 subset) at a voxel size of 0.4 × 0.4 × 0.4 mm^3^ and corrected for attenuation, scatter and decay. Images were analysed using VivoQuant (version 3.5, InviCRO) using Nucline v.0.21 software.

The data is plotted as mean ± S.D. using Graphpad 10.3.0.

## Methods

### Development of multi-inlet vortex mixer (MIVM)

An MIVM turbulent mixing apparatus was drawn based on previously reported designs,^6–8^ and electronically developed using Computer Aided Design (CAD) software. The resulting design consisted of two plates cut from PMMA using a CNC milling machine, which were bolted together as a sandwich. Screw threads were milled into the ports using a tapping tool, so that the entrance and exit ports of the mixing chip and lure-lock syringes could be connected using ETFE tubing, female luer to female adapters and flangeless male nuts in a watertight manner. Digitally controlled syringe pumps were used to provide constant and accurate flow rates for the inlet streams of the MIVM. Photographs of the mixer plates, set-up and full CAD designs can be found in the SI.

### Synthesis and characterisation of hyaluronic acid modified with dopamine (HAd) capping group

Dopamine modified hyaluronic acid (HAd) was synthesised by following a protocol by Liu *et al.* based on modified EDC/NHS chemistry.^6^ A HA (10 mg mL^−1^) solution was prepared in 400 mL distilled water, and pH adjusted to 5.0 using 1 M HCl solution. EDC (0.01 mol) and NHS (0.01 mol) were added and stirred at room temperature (r.t.), 30 min to activate the carboxylic acids of HA. Dopamine hydrochloride (0.01 mol) was added under a nitrogen atmosphere, maintained overnight (Fig. S1A). After 24 h, the solution was purified by dialysis (10K MWCO) against 18.2 H_2_O acidified with HCl (pH = 4.0) with 1.8 L water changes every 24 h for 4 days. The dialysate was measured by UV/Vis to identify the removal of free dopamine (Fig. S1B). When the absorbance of the dialysate was <0.2 a.u. after 24 h, the purified HAd was lyophilised. The degree of dopamine conjugation was determined by measuring the absorbance at 280 nm through a UV-Vis spectrometer based on a standard curve of free dopamine, and peak integration of ^1^H NMR of purified HAd dissolved in D_2_O at concentration of 10 mg mL^−1^ (256 scans) (Fig. S1C and D).

### Synthesis of FRITH-MPNPs

Solutions of HAd (1.55 mg mL^−1^), TA (0.34 mg mL^−1^), and HEPES (0.5 M, pH 5.30), were made up to 50 mL 18.2 Ω H_2_O, and 5 mL of each solution was loaded to respective syringes. FeCl_3_·6H_2_O was made up to (11.8 mM) in 2 mL water. Rapamycin (0.5 mM) was solubilised in MeOH and diluted to 0.05 mM in 18.2 Ω H_2_O. Rapamycin (2.5 mL), FeCl_3_·6H_2_O (250 µL) and ultrapure 0.1 M HCl (4.5 mL) were combined and stirred briefly to homogenously mix. 5 mL of this solution was loaded to the final syringe. The flow mixer was washed with HEPES (0.5 M, pH 5.30) prior to assembling to improve the surface wetting of the PMMA and reduce bubble formation in the mixer. The syringe pumps were programmed to 40 mL min^−1^ flow rate with a target volume of 5 mL. Three fractions (∼1 mL, 3 mL, 1 mL respectively) were collected, and the middle fraction was taken for characterisation.

### Radiolabelling FRITH-MPNPs by incubation with gallium-68

FRITH-MPNPs were purified in a PD MiniTrap™ desalting column with Sephadex G-25 resin using manufacturer's gravity protocol by loading 500 µL of FRITH-MPNPs and eluting with 700 µL PBS. The first 200 µL was discarded and the remaining 500 µL (purple colour) was collected. Gallium-68 was eluted as [^68^Ga]GaCl_3_ from two Eckert & Ziegler [^68^Ge]/[^68^Ga] generators in ultrapure 0.1M HCl. 50 µL [^68^Ga]GaCl_3_ was added to 500 µL of purified FRITH-MPNPs in PBS and stirred briefly by pipetting up and down. The solution was then purified using the gravity protocol as above, and radiochemical yield (RCY) was calculated by measuring the activity of the purified [^68^Ga]Ga-FRITH-MPNPs and column using a gamma counter.

### Preparation of [^68^Ga]Ga-FRITH-MPNPs in flow using MIVM

Solutions of HAd (1.55 mg mL^−1^), TA (0.34 mg mL^−1^), and HEPES (0.5 M, pH 5.30), were made up to 50 mL with 18.2 Ω H_2_O, and 5 mL of each solution was loaded to respective syringes. FeCl_3_·6H_2_O was made up to (11.8 mM) in 2 mL water. Rapamycin (0.5 mM) was solubilised in MeOH and diluted to 0.05 mM in 18.2Ω H_2_O. Gallium-68 was eluted as [^68^Ga]GaCl_3_ from two Eckert & Ziegler [^68^Ge]/[^68^Ga] generators in ultrapure 0.1 M HCl as 1 mL fractions. The generator eluate fractions containing >80% of the eluted radioactivity were pooled together to a combined volume of 4.5 mL (400–700 MBq). Rapamycin (2.5 mL) was mixed with FeCl_3_ (250 µL) and [^68^Ga]GaCl_3_ (4.5 mL), and 5 mL was loaded to the final syringe. The flow mixer was washed with HEPES (0.5 M, pH 5.30) prior to assembling as before. The syringe pumps were programmed to 40 mL min^−1^ flow rate with a target volume of 5 mL. Three 5 mL fractions were collected, and the middle fraction was taken for characterisation.

### Radiochemical yield of radiolabelled FRITH-MPNPs

[^68^Ga]Ga-FRITH-MPNPs were purified in a PD MiniTrap™ desalting column with Sephadex G-25 resin using the manufacturer's gravity protocol by loading 500 µL of [^68^Ga]Ga-FRITH-MPNPs and eluting with 700 µL PBS. The first 200 µL were discarded and the remaining 500 µL (purple colour) was collected. Radiochemical yield (RCY) was calculated by measuring the activity of the purified [^68^Ga]Ga-FRITH-MPNPs and column using a gamma counter. Brown-Forsythe and Welch one-way ANOVA and Dunnett T3 multiple comparisons tests were used for statistical analysis.

### Radiochemical purity of radiolabelled MPNPs

The radiochemical purity was measured by centrifuging 400 µL of a diluted suspension of the [^68^Ga]Ga-FRITH-MPNPs using ultra centrifugal filters. A small sample of the purified [^68^Ga]Ga-FRITH-MPNPs (10–20 µL) was diluted to 400 µL PBS and added to the filter, then centrifuged at a speed of 14 000 rcf for 20 min at 25 °C. Post centrifugation, only 20 µL was remaining in the filter which were diluted to 400 µL. This washing process was repeated two more times. The radioactivity of both the centrifugal filter containing [^68^Ga]Ga-FRITH-MPNPs and the combined filtrates were measured using a gamma counter. The radiochemical purity was calculated as a percentage using the eqn (1). Brown-Forsythe and Welch one-way ANOVA and Dunnett T3 multiple comparisons tests were used for statistical analysis.

Calculation for % radiochemical purity from the activity retained on an ultra-centrifugal filter and in the washes after centrifuging at 14 000 rcf, 3 min, 25 °C, with 3 PBS washes:1
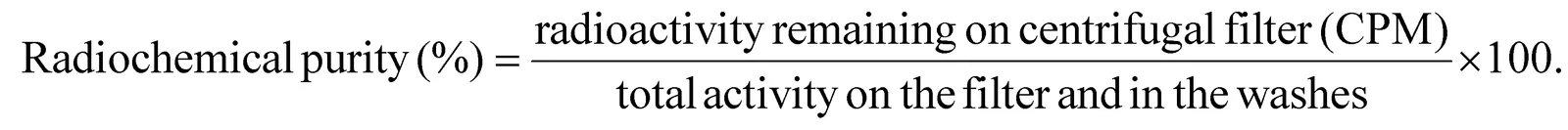


### Serum stability of radiolabelled MPNPs

Serum stability was measured by mixing [^68^Ga]Ga-FRITH-MPNPs and serum in a 1 : 1 ratio and incubated at 37 °C at a shake of 1000 rpm for 3 h. They were then filtered using the same method and calculations (eqn (1)) as for radiochemical purity. Brown-Forsythe and Welch one-way ANOVA and Dunnett T3 multiple comparisons tests were used for statistical analysis.

### Characterisation of FRITH-MPNPs and ^68^Ga-FRITH-MPNPs

#### Characterisation

The hydrodynamic diameter calculated by intensity-weighted particle size distribution, polydispersity index (PDI) and ζ-potential of the tested nanoparticles were measured at 25 °C, pH 4.4. The morphology was observed using TEM. Carbon film 300 mesh copper grids were prepared with 3 µL of FRITH-MPNPs and measured at a voltage of 120 kV. Iron(iii) concentrations in the tested samples were analysed by ICP-MS, comparing FRITH-MPNPs before and after centrifugation using a Tween 20 passivation protocol. Powder X-ray diffraction (PXRD) was used to detect diffraction peaks from the sample and determine if there is any crystallinity. Welch's *t*-test was used for statistical analysis of hydrodynamic diameter, zeta potential and % iron content.

#### Encapsulation efficiency (%EE)

Free Rapamycin was isolated using centrifugal filters at a speed of 14 000 rpm for 20 min at 25 °C. A passivation method using Tween 20 to prevent rapamycin sticking to the filters was used. The method based on the manufacturers protocol^27–29^ to improve rapamycin filtering through centrifugal filters was as follows: 400 µL of 5% Tween-20 was added to a centrifugal filter and left at r.t. After 1 week, the filter was emptied and washed with H_2_O (18.2 Ω). To remove residual solution, the filter was filled with 400 µL of H_2_O (18.2 Ω) and centrifuged (14 000 rcf, r.t., 10 min) three times. The filter was inverted and centrifuged (1000 rcf, r.t., 3 min) to remove the remaining water. 400 µL [^68^Ga]Ga-FRITH-MPNPs mixed with 400 µL EtOH (10% v/v) (to ensure solubilisation of free rapamycin) was added to the washed filter, and centrifuged (14 000 rcf, r.t., 20 min).

The free rapamycin level in the filtrate after centrifugation was measured using reverse phase HPLC with a C-18 column. The HPLC method used for measuring encapsulation efficiency was as follows: solvent A = water (0.1% formic acid v/v); solvent B = methanol (0.1% formic acid v/v); gradient as per Table 1; injection volume 20 μL; column temperature 40 °C; wavelength 278 nm; instrument: Agilent 1260 Infinity II HPLC; column: Agilent Poroshell 120 EC-C18, pore size 2.7 μm, internal diameter 4.6 mm, length 50 mm.

**Table 1:** HPLC method used for the determination of rapamycin encapsulation in FRITH-MPNPs

Time/min	Flow rate/mL min^−1^	%A	%B
0	1	95	5
1	0.5	95	5
2	0.5	20	80
12	0.5	5	95
13	0.5	5	95
14	0.5	95	5
17	0.5	95	5

The drug encapsulation efficiency (EE, %) of rapamycin in the FRITH-MPNPs was back calculated from a rapamycin calibration curve and using eqn (2). Welch's *t*-test was used to statistical analysis.

Calculation for encapsulation efficiency from concentrations obtained by HPLC and theoretical concentration from maximum possible concentration added in synthesis:2
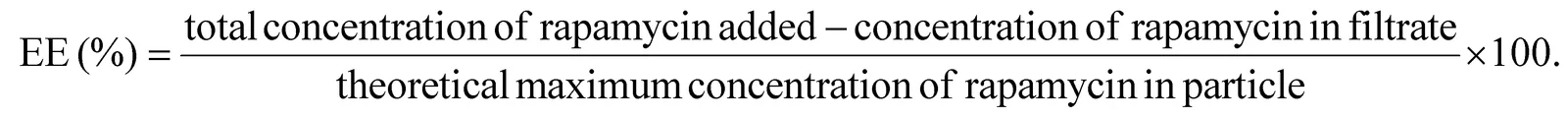


### Cell culture

Mouse monocyte macrophages J774A.1 cells were cultured in Dulbecco's modified Eagle's High Glucose medium (DMEM) containing 10% fetal bovine serum (FBS), 1 mM sodium pyruvate, 2 mM l-glutamine and 6 mL (1%) penicillin–streptomycin at 37 °C under a humidified atmosphere with 5% CO_2_. J774A.1 cells were harvested by physical scraping method for the experiments in this study.

### *In vitro* cytotoxicity assay

The cytotoxicity of the FRITH-MPNPs was determined by a WST-8 assay performed on J774a.1 cells. Cells were harvested before reaching 80% confluency *via* physical scraping method for J774a.1. The harvested cells were resuspended in 10 mL media and centrifuged at 500 rcf, 3 min. The pellet was resuspended in 5 mL media. Cell seeding density was determined after a calibration curve was carried out at 24 and 48 h between 3000–50 000 cells per well (Fig. S6). Cell plating was carried out at a density of 5000 cells per well in a 96-well clear bottom plate and allowed to adhere 24 h, 37 °C, 5–10%, CO_2_. The FRITH-MPNPs were 10× serially diluted using media with 10% PBS to concentrations of 10%, 1%, 0.1% and 0.01% particles. The old media was removed and 200 µL of each complex was added to the plates and incubated 37 °C, 5–10%, CO_2_. The positive control was untreated cells in 10% PBS, where the old media was removed and media with 10% PBS was added in place of FRITH-MPNPs. A negative control was run with FRITH-MPNPs without cells to ensure the colour of the particles does not interfere with the colorimetric output of the WST-8 assay. After 24, 48 and 72 hours 10 µL WST-8 solution was added and incubated for 4 hours 37 °C, 5–10% CO_2_. The plates were read using a microplate reader at 450 nm. The cell viability of each well was normalised against the untreated control wells. Kruskal–Wallis one-way ANOVA and Dunn's repeated measures tests were used to statistically analyse *in vitro* data.

### *In vivo* PET/CT imaging

All animal procedures and imaging protocols were ethically reviewed and approved by the local Animal Welfare and Ethical Review Body (AWERB) under UK Home Office Project Licence Number PBBA9A243. All work was performed in strict accordance with the Animals (Scientific Procedures) Act 1986 (ASPA). *In vivo* imaging was conducted in healthy 9-week-old BALB/c mice (*n* = 3). Animals were anaesthetised with isofluorane with oxygen flow of 1 L min^−1^, cannulated and placed on the scanner 4-mouse hotel bed under anaesthesia. The respiration rate was monitored throughout the scan. The PET acquisition was started and 150 µL containing 0.36–0.37 MBq of [^68^Ga]Ga-FRITH-MPNPs (*n* = 3) was administered through the cannula, followed by A wash with 50 µL PBS. PET/CT images were acquired using a PET/CT scanner. PET was acquired for 4 h, (1 : 5 coincidence mode; 5 ns coincidence time window) followed by a CT scan. Dynamic PET/CT images were reconstructed at times interval 0–15 min, 15–30 min, 30–60 min, 60–90 min, 90–120 min, 120–180 min and 180–240 minutes.

The free [^68^Ga]Ga^3+^ control was carried out using BALB/c mice (*n* = 3). [^68^Ga]GaCl_3_ from an Eckert & Ziegler [^68^Ge]/[^68^Ga] generator in ultrapure 0.1M containing 10 MBq was neutralised with 1 M sodium carbonate to ∼pH 7. This was then passed through a pre-rinsed centrifugal filter (MW cutoff 50 kDa) to remove potential colloids, using saline to wash the filter and to ensure all free [^68^Ga] was collected in the filtrate. At this point, 100 µL of the filtrate, containing colloid-free [^68^Ga] was administered *via* IV injections. A dynamic PET scan was acquired for 2 h after injection followed by a CT scan.

PET/CT images were analysed by drawing ROIs and the data was pre-processed to show the ROIs as percentage injected activity (activity in the ROI/total activity injected) per gram of tissue (% IA per g). All images for both the free [^68^Ga]Ga^3+^ control and [^68^Ga]Ga-FRITH-MPNP biodistribution are shown on the same scale to ensure comparisons can be drawn.

### *Ex vivo* biodistribution

Uptake in different organs was evaluated by gamma counting. After the *in vivo* PET/CT imaging, animals were culled by cervical dislocation and organs of interest were excised and weighed. The organs were counted in a gamma counter. Data were expressed as percentage injected activity (activity in the organ/total activity injected) per gram of tissue (% IA per g).

## Results

### Development of the multi-inlet vortex mixer (MIVM) and optimised synthesis of FRITH-MPNPs

**Fig. 2:**
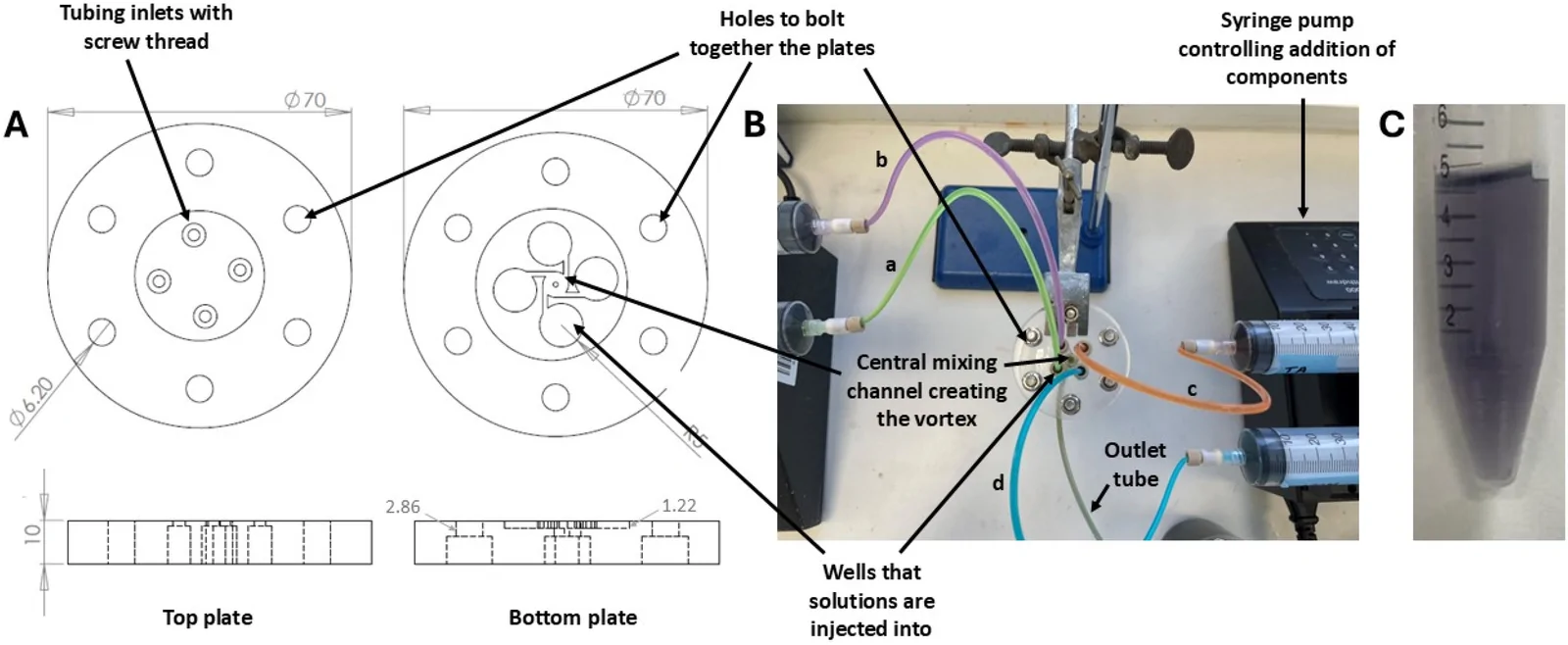
Flow mixer development (A) CAD design of top and bottom plates detailing the dimensions for the MIVM mixer, full CAD drawings and dimensions can be found in SI; (B) MIVM flow mixing set-up including syringe pumps and 50 mL syringes and ETFE tubing that connects to the MIVM mixer. Food colouring is used here for visualisation purposes, in the experiment the tubes labelled (a) tannic acid, (b) Fe(iii) + rapamycin, (c) HEPES, (d) Hyaluronic acid modified with dopamine (HAd); (C) photograph of FRITH-MPNPs showing purple colour, indicating formation of MPN.

FRITH-MPNPs were synthesised by a flow synthesis method using a multi-inlet vortex mixer (MIVM) with four inlets for each of the respective components: hyaluronic acid modified with dopamine (HAd) (details of synthesis and degree of modification is shown in SI, Fig. S1), Tannic Acid (TA), iron pre-mixed with rapamycin, and HEPES buffer, which controls the pH of the reaction. The syringe pumps were programmed to 40 mL min^−1^ and the required volume was easily produced by filling the syringes to the appropriate volume. The resultant suspension was visibly purple in colour (Fig. 2C).

### Radiolabelling of [^68^Ga]Ga-FRITH-MPNPs

FRITH-MPNPs were radiolabelled with gallium-68 *via* two methods: a post-synthesis method where the particles were synthesised and purified before incubation with the gallium-68, followed by a novel method, where the gallium-68 is added to the MIVM, enabling simultaneous synthesis and radiolabelling of nanoparticles for PET imaging in one-step.

**Fig. 3:**
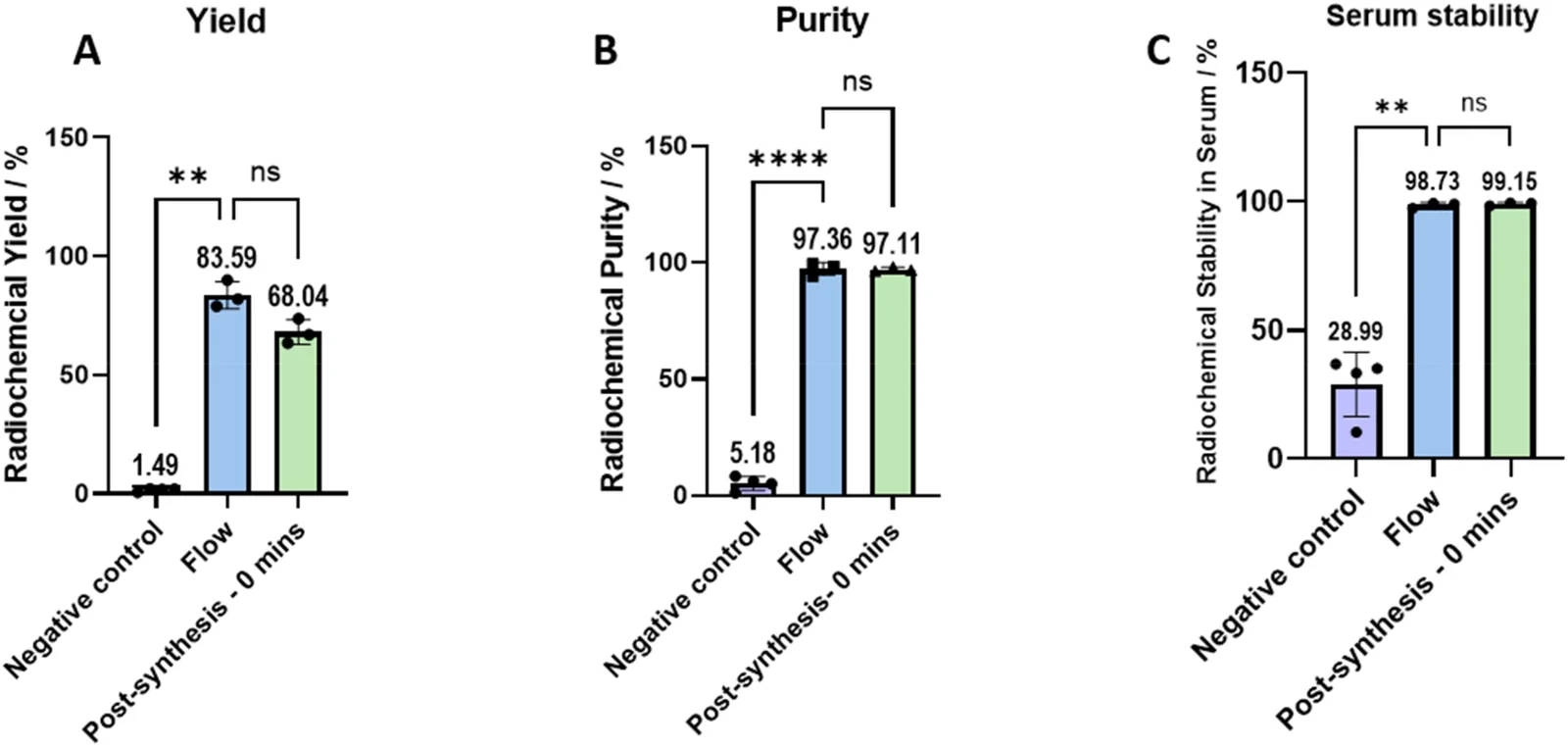
Radiolabelling data: (A) Radiochemical yield of galllium-68 bound to FRITH-MPNPs using radiolabelling by flow and post-synthesis radiolabelling methods (*n* = 3), compared with a control of free gallium-68 in PBS; (B) radiochemical purity of [^68^Ga]Ga-FRITH-MPNPs (*n* = 3) labelled by a flow method and post-synthesis radiolabelling method, compared with a control of free gallium-68 in PBS; (C) radiochemical stability of [^68^Ga]Ga-FRITH-MPNPs in serum (*n* = 3), labelled by a flow method and post-synthesis radiolabelling method, compared with a control of free gallium-68 in PBS mixed in a 1 : 1 ratio with serum. Asterisks represent statistical significance of the *p*-value where: ** *p* < 0.01; **** *p* < 0.0001; ns *p* ≥ 0.05.

The radiolabelling yield of the [^68^Ga]Ga-FRITH-MPNPs was determined using a size exclusion column, where unincorporated free [^68^Ga]Ga^3+^, as well as gallium-68 associated to any of the other FRITH-MPNPs components was trapped in the column. The difference in activity between the column and purified [^68^Ga]Ga-FRITH-MPNPs in PBS was then measured in a dose calibrator or gamma counter to calculate the radiochemical yield of gallium-68 labelling to the particles. [^68^Ga]Ga-FRITH-MPNPs that were labelled by directly adding gallium-68 to the particles post-synthesis had a radiolabelling yield of 68.0 ± 5.2%, whilst [^68^Ga]Ga-FRITH-MPNPs labelled by incorporation of the radionuclide into the flow synthesis was 83.6 ± 4.7% (Fig. 3A).

Radiochemical purity was measured using centrifugal size exclusion filters. [^68^Ga]Ga-FRITH-MPNPs labelled post-synthesis exhibited a purity of 97.1 ± 0.9% and when the gallium-68 is added to the flow synthesis, the purity was 97.4 ± 2.6% (Fig. 3B).

Serum stability was tested using the same method as for purity after incubating the [^68^Ga]Ga-FRITH-MPNPs in a 1 : 1 ratio with serum for 3 hours. When radiolabelling the particles after synthesis, the radiochemical stability in serum was 99.2 ± 0.6%, and when radiolabelled by flow was 98.7 ± 1.1% (Fig. 3C). The negative control of serum incubated with [^68^Ga]GaCl_3_ shows 30% of activity retained in the ultracentrifugation filter due to the filter material and pore size not being sufficient to separate [^68^Ga]Ga-FRITH-MPNPs from the serum proteins completely. Despite this, the value obtained from the control experiment is significantly different from both the flow and post synthesis values, giving confidence that [^68^Ga]GaCl_3_ is not showing a greater affinity for serum proteins over Ga-FRITH-MPNPs.

### Characterisation of FRITH-MPNPs and ^68^Ga-FRITH-MPNPs

**Fig. 4:**
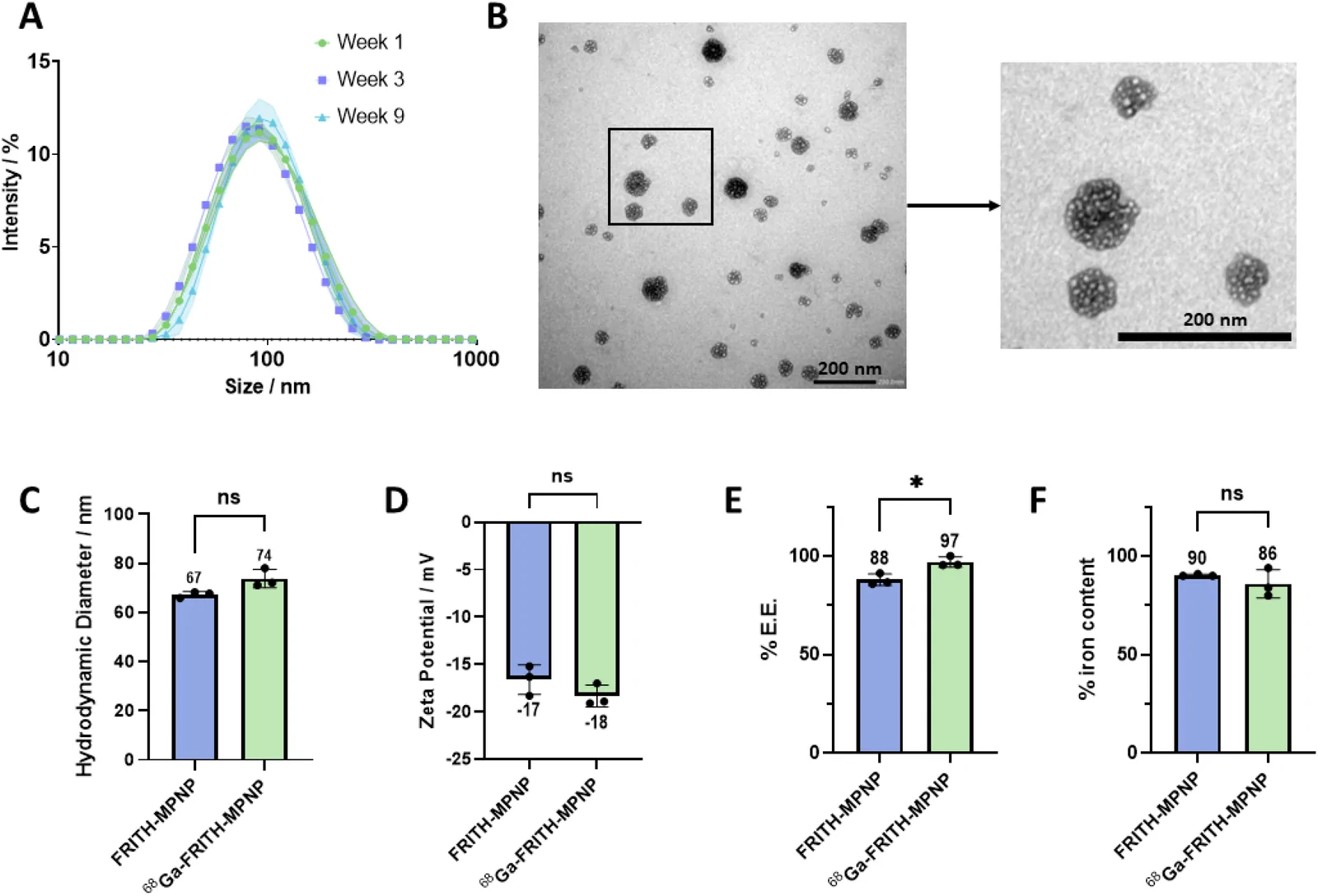
Nanoparticle characterisation (A) DLS data showing hydrodynamic diameter of FRITH-MPNPs directly after synthesis, 4 and 9 weeks later; (B) transmission Electron Microscopy images of FRITH-MPNPs, scale bar 200 nm; (C) DLS data showing the hydrodynamic diameter of unlabelled FRITH-MPNPs and [^68^Ga]Ga-FRITH-MPNPs; (D) zeta potential data showing the difference in surface charge between unlabelled FRITH-MPNPs and [^68^Ga]Ga-FRITH-MPNPs; (E) ICP-MS data showing metal content in [^68^Ga]Ga-FRITH-MPNPs and FRITH-MPNPs (*n* = 3), including a blank of iron bound to rapamycin in the same ratios as expected in the resulting particles; (F) encapsulation efficiency of rapamycin in FRITH-MPNPs back-calculated from the filtrate ultracentrifuged FRITH-MPNPs.

The [^68^Ga]Ga-FRITH-MPNPs labelled *via* the flow method had the most optimal radiolabelling and were chosen for further characterisation to ensure that the radiolabelling did not impact the structure and properties of the particles. Hydrodynamic diameter of both FRITH-MPNPs and [^68^Ga]Ga-FRITH-MPNPs was measured using dynamic light scattering (DLS). The average hydrodynamic diameter (mean ± SD) was 67 ± 1 nm and PDI 0.205 ± 0.02 for FRITH-MPNPs, and 74 ± 3 nm with PDI 0.232 ± 0.02 for [^68^Ga]Ga-FRITH-MPNPs, and measurement over a period of 9 weeks showed minimal change in size, indicating colloidal stability (Fig. 4A & C). The surface charge was measured using zeta potential measurements to determine if the radiolabelling changes the surface properties. FRITH-MPNPs had a zeta potential of −16.6 ± 1.3 mV and [^68^Ga]Ga-FRITH-MPNPs had a surface charge of −18.3 ± 0.95 mV (Fig. 4D). The size of the particles was confirmed by TEM, (Fig. 4B), with the average particle size for FRITH-MPNPs being 70 ± 42 nm (analysed with ImageJ, *n* = 50). The large dispersity seen in the image, and reflected in the standard deviation of the TEM-based size measurements, owes to the particle structure being based on a water exchange mechanism, leading to fluctuation in size when subjected to vacuum, preparing for TEM analysis. As a result, it is likely that DLS produces a more representative value for diameter of FRITH-MPNPs. A HPLC method was developed to measure the rapamycin encapsulation efficiency (EE). The FRITH-MPNPs were separated from any unbound drug using centrifugal size exclusion filters combined with a Tween 20 passivation protocol. The filtrate was analysed by HPLC, and the concentration of unbound rapamycin was determined using a calibration curve (Fig. S2). This value was subtracted from the total theoretical rapamycin concentration, resulting in the calculated concentration of rapamycin encapsulated within the FRITH-MPNPs. The encapsulation efficiency (EE) for [^68^Ga]Ga-FRITH-MPNPs was calculated to be 97 ± 2%, whilst FRITH-MPNPs that were not radiolabelled had an EE of 88 ± 2% (Fig. 4E). The mass content of iron in the particles was measured using ICP-MS by measuring the difference in metal content between a sample filtered using the centrifugal size exclusion method and an unfiltered sample. The iron measured in the [^68^Ga]Ga-FRITH-MPNPs was 85.612 ± 0.059% and FRITH-MPNPs was 90.217 ± 0.004%. An unpaired *t*-test was performed on the radiolabelled and non-radiolabelled samples and deemed not significant (Fig. 4F). PXRD was used to determine whether the FRITH-MPNPs is crystalline. Unfortunately, PXRD confirmed the amorphous nature of the MPN coordination network, precluding further structural characterisation by diffraction methods.

### *In vitro* cell toxicity

**Fig. 5:**
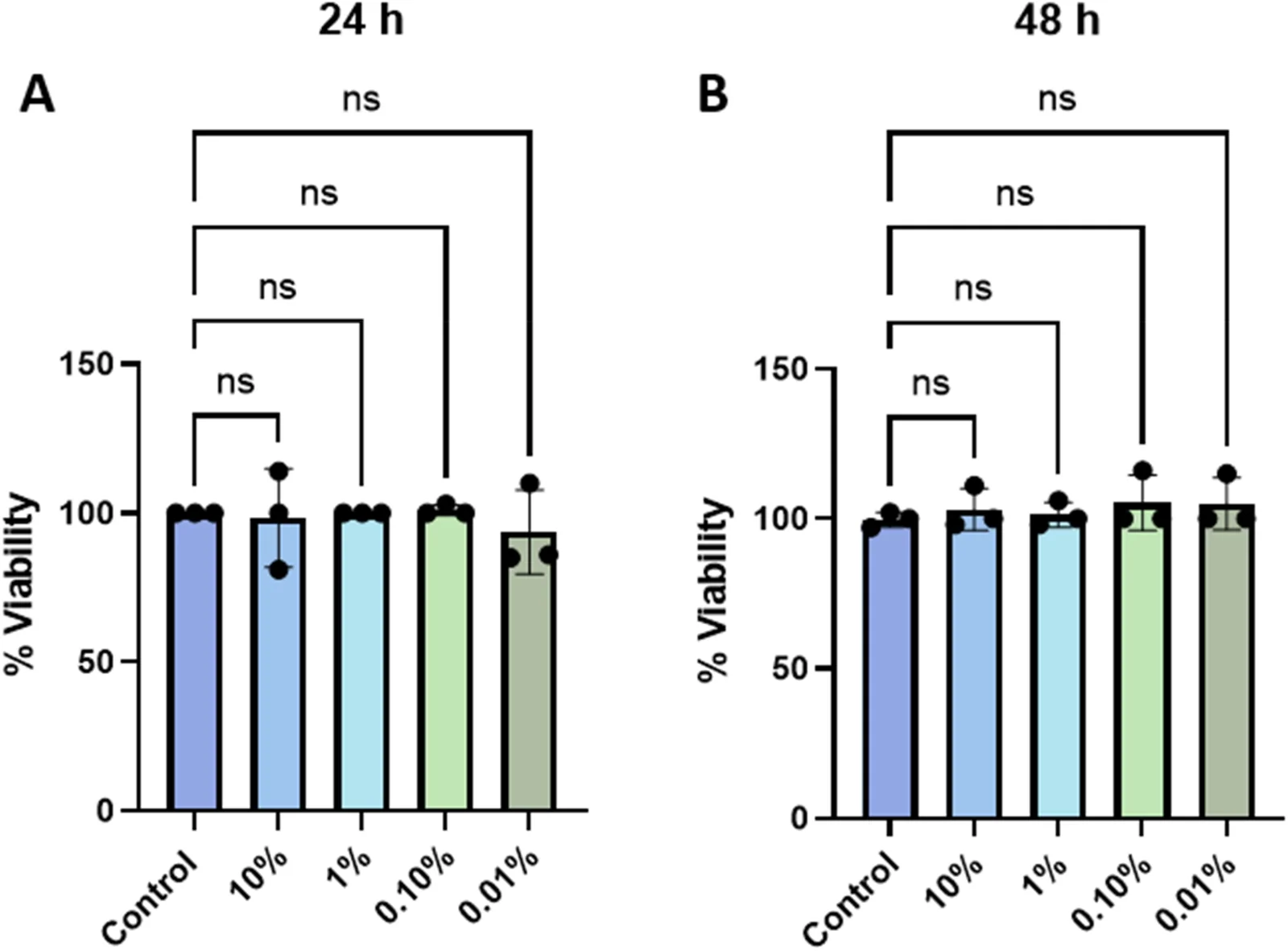
Cell viability study on J774a.1 murine macrophage cell line using WST-8 reagent (A) 24 h and (B) 48 h after treatment of cells with particles.

The cell viability of FRITH-MPNPs was tested on a macrophage cell line to determine how safe the particles would be *in vivo*. After diluting in PBS, FRITH-MPNPs were added to J774a.1 macrophages. A one-step water soluble assay for determining cell viability and cytotoxicity (WST-8), was used to measure the viability after incubating the cells with FRITH-MPNPs for 24 h and 48 h. The results (Fig. 5) show that there was no cell death at either time point, even at the highest particle concentration, compared with a control of cells in 10% PBS in media.

### *In vivo* PET/CT imaging & *ex vivo* biodistribution

**Fig. 6:**
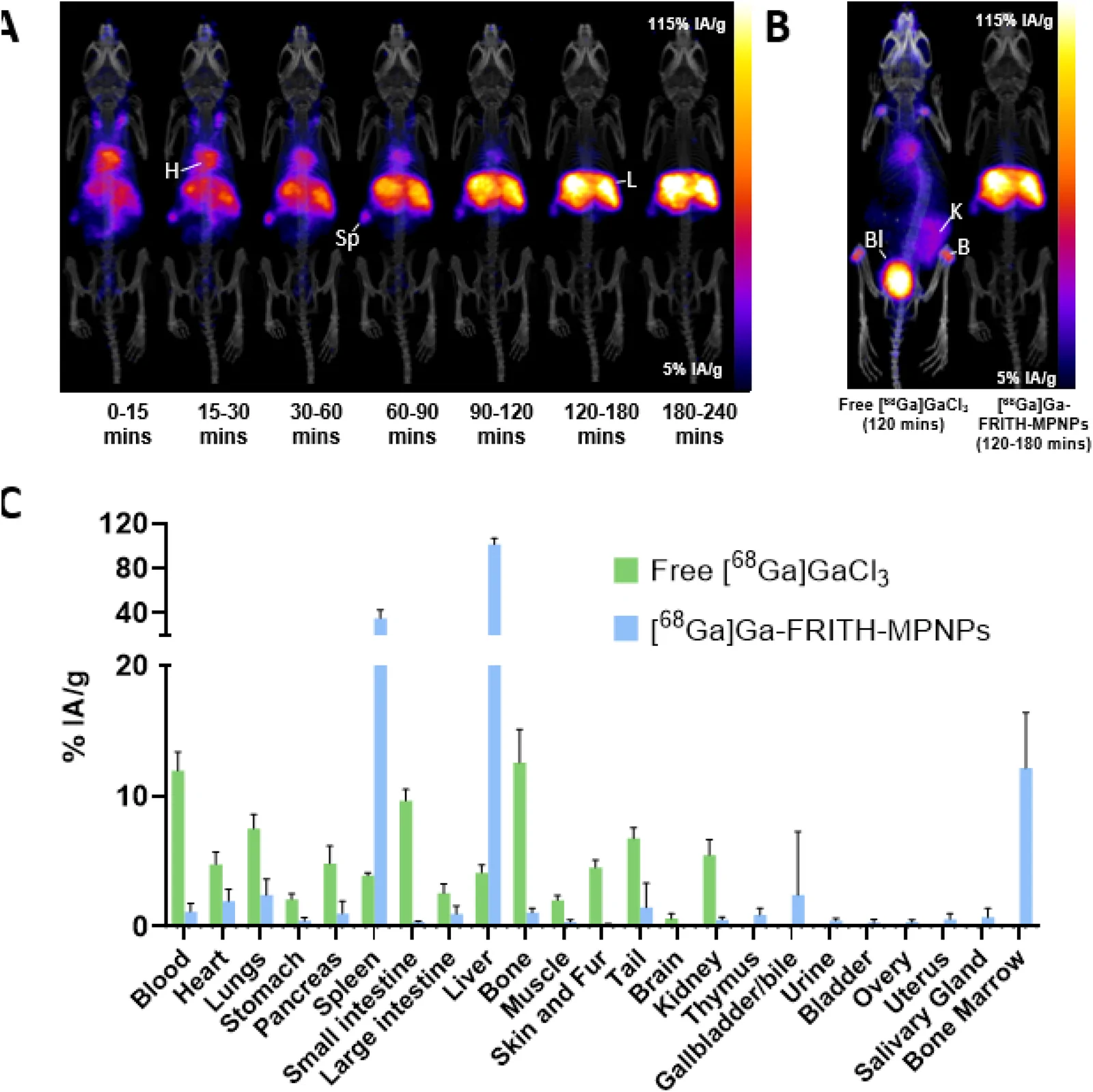
PET Imaging (A) PET-CT images of a healthy BALB/c mouse injected with [^68^Ga]Ga-FRITH-MPNPs across a 4 h scanning period showing blood circulation time of 3 h and beginning in the heart (H) and finally accumulating in liver (L) and spleen (Sp); (B) PET-CT image of a healthy BALB/c mice injected with free gallium-68 over 2 h scanning period as a control, showing uptake in the bladder (Bl), kidney (K) and bones (B) (left), compared with a BALB/C mouse injected with [^68^Ga]Ga-FRITH-MPNPs between 120–180 min; (C) *ex vivo* biodistribution data showing organ activity after 4 h, showing accumulation in liver, spleen and bone marrow, and control with free [^68^Ga]Ga^3+^ after 2 h, showing uptake in blood, bone and small intestine.

After successful radiolabelling, the biodistribution and radiolabelling stability of the [^68^Ga]Ga-FRITH-MPNPs was assessed *in vivo* using PET imaging. [^68^Ga]Ga-FRITH-MPNPs were injected intravenously into healthy mice, and a four-hour dynamic PET scan was immediately carried out. *Ex vivo* biodistribution was performed on all mice after the imaging sessions. PET/CT imaging of the mice showed blood pool circulation for the first 15 min, followed by increasing uptake in the liver and spleen (Fig. S7). After 3 h the majority of [^68^Ga]Ga-FRITH-MPNPs were taken up in the liver (101.10 ± 0.04% ID per g, *n* = 3) and spleen (35.12 ± 0.06% ID per g, *n* = 3). Compared to a control of free gallium, [^68^Ga]Ga-FRITH-MPNPs showed an absence of bone uptake and there was no renal excretion (Fig. 6B). As the radioisotope concentrations are in tracer amounts, differences in biodistribution of free [^68^Ga]GaCl_3_ are not expected with decreasing activity to that used for the [^68^Ga]Ga-FRITH-MPNPs. Therefore, the biodistribution images verify that [^68^Ga]Ga-FRITH-MPNPs are stable, with no observable free [^68^Ga]Ga^3+^ dissociating from the particles. This is confirmed by *ex vivo* biodistribution showing final accumulation predominantly in the liver, spleen and bone marrow (Fig. 6C).

## Discussion

It was hypothesised that radiolabelling of FRITH-MPNPs in flow will result in radiometal binding in the same fashion as iron to the polyphenol, becoming part of the particles’ coordination network. Adding trace amounts of radionuclide into the metal solution prior to a flow synthesis rather than combining it with the pre-formed nanoparticle post-synthesis, should result in the radionuclide being strongly bound between the polyphenols, leading to a higher yield and stable radiolabelling. Furthermore, it would enhance the labelling by employing the full particle volume, whilst leaving the surface unaltered for further targeting and functionalisation.^10^

Metal-phenolic networks (MPNs) were assembled by coordination between Fe(iii) and TA to form a particle core. A therapeutic can then be trapped in this network and the particle core is then surrounded by a polymer which can be utilised for functionalisation and targeting.^6^ It is established in the literature that therapeutics can be released from MPN networks; this is especially accelerated in tissues where pH is reduced.^12^ In the particles reported in this work, the incorporated therapeutic was rapamycin, and the particles were surrounded by hyaluronic acid modified with dopamine (HAd), which enables metal binding to the MPN core. These complex particles, which we call FRITH-MPNPs (flow-synthesised rapamycin, iron(iii), tannic acid and HAd containing metal–phenolic nanoparticles) are formed in a one-step process using an MIVM, which is a flow mixer with four ports, connected to syringe pumps, allowing direct, controlled and homogeneous mixing of the components.

The MPNs in this study were made from TA due to its known biocompatibility and FDA approval, and Fe(iii) was chosen due to accessibility and extensive fundamental understanding of its behaviour and properties.^30,31^ The therapeutic must have a carbonyl, hydroxyl or other functional groups with the ability to bind to the MPN network in its structure in order to interact with Fe(iii), as well as proven use for a variety of applications *in vivo*. Rapamycin (also known as Sirolimus) was selected due to its wide ranging potential applications including as an immunosuppressant for organ transplant,^32^ anti-aging^33^ and cancer.^34,35^

The resulting particles have a distinct purple colour, thanks to the well-documented metal–ligand bonding interactions that occur in MPNs. At pH 5, the approximate pH at which this assembly takes place, the more acidic hydroxyl groups of tannic acid are able to donate their π-electrons into the δ-orbitals of the Fe(iii) centre, creating a distorted octahedral or tetrahedral geometry allowing a bis-coordinated geometry of two ligands to one metal centre. The reduction in orbital energy gap causes enhanced ligand metal charge transfer (LMCT) transitions, resulting in a purple-coloured solution, which in turn is indicative of MPN formation.

Belonging to the period 4 of the periodic table, iron(iii) and gallium(iii) have similar ionic radii and essential chemical properties including the same coordination number and geometry.^36,37^ To this end, gallium-68 was chosen as an appropriate radiometal to incorporate as a replacement for iron(iii). Furthermore, gallium-68 is an attractive choice on account of production *via* a clinical grade on-site benchtop generator as opposed to a cyclotron.^38^ This allows the gallium-68 isotope to be highly accessible at low cost, requiring less space compared to a cyclotron and can be implemented on-site in a clinical setting.

The flow mixer was based on previous designs,^7,8^ but adapted for the purposes of this work, incorporating a outlet channel to direct the [^68^Ga]Ga-FRITH-MPNPs to a shielded container. Additionally, it was assembled from transparent PMMA rather than steel to allow visualisation inside the mixer at all times. This meant that any leaks which would lead to radiation contaminations, as well as any bubbles forming in the mixer, which may impact successful FRITH-MPNPs formation, could be observed. Additionally, PMMA is easy to work with, as it is affordable and no additional protective equipment is required other than the CNC milling machine to produce the mixer. Successful implementation of the mixer was established by comparing conventional mixing methods with the flow method, obtaining comparable results to literature,^6^ with flow assembled particles producing a smaller hydrodynamic diameter than with conventional syntheses. This is because controlling the flow rate of the reagent addition enables faster, more controlled, mixing, resulting in high supersaturation that promotes rapid nucleation, while limiting subsequent growth and preventing aggregation.^8^

When implementing the post-synthesis radiolabelling method by adding the [^68^Ga]GaCl_3_ after synthesis, with no incubation time, the radiochemical yield was 68.0 ± 5.2%. This high yield combined with the high radiochemical purity and serum stability is a testament to the strong metal coordination that can occur on the particle surface.

The novel radiolabelling method being explored in this work is to directly add the radiometal into the flow synthesis. Gallium-68 was easily added to the syringe containing the iron hexahydrate, and the synthesis was carried out as with the non-radioactive synthesis, requiring no further purification or neutralisation steps, or complicated complexation as would be required with other kinds of radiolabelling. By doping into the iron-rapamycin mixture, the high millimolar concentration of iron can be kept far in excess of the picomolar concentrations of gallium (nearly 2 million times more iron than gallium), so that whilst the metals are similar, there should be minimal further impact on any potential metal binding or other structural changes. Incorporating the radioisotope into the flow synthesis elevated the radiochemical yield to 83.6 ± 4.7%. This suggests that incorporating gallium-68 into the flow synthesis enabled coordination to more metal binding sites in the core of the particle than are accessible solely on the surface, enhancing the radiochemical yield. The radiochemical purity and serum stability were very high and repeatable, with an insignificant difference between the two radiolabelling methods according to ANOVA analysis, indicating that radiolabelling during the flow synthesis did not impede the ability for gallium-68 to strongly bind and become part of the MPN particle structure.

Zeta potential measurements were used to study the effect of radiolabelling on the surface charge of the particles. Non-labelled FRITH-MPNPs had a surface charge of −16.6 ± 1.28 mV and radiolabelled [^68^Ga]Ga-FRITH-MPNPs −18.33 ± 0.95 mV, showing a negligible difference and suggesting that the surface is free to be functionalised for targeting, as opposed to forming a metal shell.^39,40^ Additionally, a slightly negative zeta potential is beneficial for the *in vivo* stability of particles, reducing aggregation and interactions with protein corona. Further characterisation techniques show that [^68^Ga]Ga-FRITH-MPNPs radiolabelled in the flow synthesis have similar properties to the FRITH-MPNPs which were not labelled. DLS measurements show that the hydrodynamic size of the particles varies by between 67 ± 1 nm for [^68^Ga]Ga-FRITH-MPNPs and 74 ± 3 nm for unlabelled FRITH-MPNPs, which is a small size difference when considering how size affects biodistribution *in vivo*.^41,42^ In addition to corroborating DLS measurement of particle size, TEM shows particle morphology. We observe ‘berry-like’ shape, similar to previous reports (though these are shown at lower resolution),^6^ confirming successful synthesis. This morphology may reflect the effect of drying and electron beam damage, or that the particles are formed by association of smaller subunits making up the larger particles (analogous to formation of ‘nanoflowers’, iron oxide nanocrystals aggregating into larger secondary particles when coated with poly acrylic acid),^43–46^ perhaps mediated by nucleation at HAd polymer, and warrants further investigation beyond this study.

The iron content between the [^68^Ga]Ga-FRITH-MPNPs and unlabelled FRITH-MPNPs varied from 85.612 ± 0.059% to 90.217 ± 0.004% respectively. The insignificant difference in iron content between the two groups as determined by an unpaired *t*-test, as well as the characteristic purple colour observed upon formation, further corroborates that the metal coordination and geometry is not impacted by the introduction of radiometal. Furthermore, the picomolar concentrations of gallium-68 (*versus* millimolar concentrations of iron) means that the impact on the concentration ratios of other components is negligible. Encapsulation efficiency calculated by HPLC was 96.98 ± 2.14% for [^68^Ga]Ga-FRITH-MPNPs and 87.96 ± 2.36% for unlabelled FRITH-MPNPs. This was found to be a significant difference and thought to be due to the introduction of radiation to the particles.

The *in vitro* cytotoxicity assay was used to measure several different particle concentrations on J774a.1 macrophages over 24 h and 48 h. Macrophages were used as the size and surface charge of the FRITH-MPNPs give confidence that in a healthy murine model, uptake would be seen predominantly in the liver and spleen as a result of MPS uptake. At both time points it was determined using ANOVA analysis that cell death was negligible for every particle concentration administered to the cells, and it was concluded that it would be safe to proceed with *in vivo* studies.

Intravenous injection of [^68^Ga]Ga-FRITH-MPNPs into a healthy mice showed uptake primarily in the liver, spleen and bone marrow based on the PET/CT images and *ex vivo* biodistribution after 4 h. This time point was chosen due to the short half-life of gallium-68, and the expected circulation time based on recent reports with nanoparticles of similar physicochemical properties.^6^ This biodistribution was expected for nanoparticles of this size in healthy mice.^47^ The biodistribution of [^68^Ga]Ga-FRITH-MPNPs was in contrast to the control of free [^68^Ga]GaCl_3_, where excretion of the radioisotope was observed through the renal system, with areas of high activity in the kidney, bladder and bones after only 2 hours. In contrast, for the [^68^Ga]Ga-FRITH-MPNP biodistribution over 4 hours, the kidney was not observed by PET, suggesting no renal excretion. The *ex vivo* biodistribution data further verified this observation with 0.558 ± 0.002% IA per g measured in the kidney compared to 5.455 ± 0.979% IA per g in the free gallium control. Furthermore, no appreciable bone uptake in the [^68^Ga]Ga-FRITH-MPNPs group was observed with 1.063 ± 0.003% IA per g measured in the *ex vivo* biodistribution, compared with 12.534 ± 2.079% IA per g in the free gallium-68 control. The free gallium-68 group showed significantly less uptake in the spleen (3.908 ± 0.173% IA per g control *vs*. 35.121 ± 0.059% IA per g [^68^Ga]Ga-FRITH-MPNPs) and liver (4.121 ± 0.479% IA per g control *vs*. 101.097 ± 0.043% IA per g [^68^Ga]Ga-FRITH-MPNPs) than the [^68^Ga]Ga-FRITH-MPNPs. Furthermore, there was clear uptake of [^68^Ga]Ga-FRITH-MPNPs in the bone marrow, which is an important feature for future use in exciting new applications such as trained immunity targeting.^48^ The differences between the two groups confirmed that the imaging features observed in the [^68^Ga]Ga-FRITH-MPNP group are due to particle circulation in the body and accumulation in organs of the MPS. The lack of kidney and bone uptake indicates no free gallium circulating with the particles, thereby confirming stable radiolabelling of [^68^Ga]Ga-FRITH-MPNPs, and hence making these radiolabelled particles highly suitable for use as an imaging guided drug delivery agent.

Several MPN-based nanoparticles have previously been adapted for PET imaging.^12–19,49,50^ Guo *et al.* synthesised Cu(ii)-TA particles by bulk mixing the MPN with a polystyrene substrate, which was later dissolved to yield a hollow capsules.^12^ Rigorous characterisation using UV/Vis, FTIR, and XPS confirmed metal coordination, while DIC, SEM, TEM, and AFM showed the capsules were monodisperse and spherical. The radiometal [^64^Cu]Cu was incorporated into the thin film shell during assembly, effectively doping the non-radioactive copper. A PET phantom image confirmed the suitability for biodistribution tracking *in vivo*. As in our study, healthy BALB/c mice were intravenously injected and scanned for 30 min. PET image quantification showed no cardiac uptake, suggesting MPS (mononuclear phagocyte system) clearance dominated over passive EPR-based tumour targeting. *Ex vivo* analysis revealed uptake in the liver, spleen, and kidney. Owing to missing urine excretion data, comparison with our 30 min dataset was not possible. Consistent with our free gallium-68 control, kidney uptake likely reflects low *in vivo* radiolabelling stability and free radiometal accumulation. This comparison indicates that flow mixing synthesis and polymer capping enhance radiolabelling stability and extend particle half-life for passive targeting.

Suárez-Garcia *et al.* also formed two MPN particles from caffeic acid, 1,4-Bis(imidazole-1-ylmethyl)-benzene and [^64^Cu]CuCl_2_, coated with PEG.^16^ Characterisation by SEM, EDX, FTIR, zeta potential, DLS, ICP-MS, XPS, ^1^H-NMR, and PXRD showed a size range of 119–127 nm and surface charge of −30 mV. Despite high radiochemical yield (87%) and purity (>95%), intravenous administration in BALB/c mice caused acute respiratory distress within 15 minutes, ending the study and suggesting *in vivo* aggregation. A repeated trial with fewer nanoparticles allowed *ex vivo* biodistribution quantification after 5 h, revealing uptake mainly in lungs and blood, and lower levels in liver, intestine, kidneys, spleen, and tumour. Kidney uptake again suggested partial *in vivo* isotope release, and distribution variability may relate to the more negative surface charge. Nonetheless, tumour uptake, likely due to the EPR effect, indicates partial MPS evasion in this tumour model.

The FRITH-MPNPs and MIVM flow mixer in this work were adapted from Liu *et al.*, with further modifications for PET imaging.^6^ Characterisation revealed strong similarities to both [^68^Ga]Ga-FRITH-MPNPs and unlabelled FRITH-MPNPs despite the different composition. In Liu's study, tumour uptake was imaged *via* MRI 12 h post-injection in an MCF-7 xenograft model, showing enhanced T1-weighted signals. Doxorubicin-containing particles served as MR contrast agents for image-guided therapy. *Ex vivo* fluorescence imaging confirmed uptake in lungs, kidneys, spleen, liver, and tumour, demonstrating MPS evasion and potential for passive drug delivery. Notably, higher kidney and lung uptake than in spleen and liver may reflect premature doxorubicin release or aggregation from insufficiently negative surface charge or incomplete capping. In contrast, our [^68^Ga]Ga-FRITH-MPNPs showed exclusive liver and spleen uptake with no renal or pulmonary accumulation, indicating exceptional *in vivo* stability. Thus, FRITH-MPNPs have been effectively translated into robust PET imaging agents with whole-body imaging capability.

This method of directly labelling an MPN nanoparticle core with the short-lived radiometal gallium-68 in a one-step synthesis is shown to have high *in vivo* stability and practical applications to medical imaging with PET. Whilst the biodistribution of [^68^Ga]Ga-FRITH-MPNPs in this work has only been studied in a healthy model, previous reports show that similar MPN-based nanoparticles can be taken up by solid tumours in disease models, indicating that it could be possible to overcome the mononuclear phagocyte system (MPS), especially with the addition of a stealth polymer coating such as PEG.^51^ It would also be prudent to modify the MIVM mixer into a fully automated system, enabling a transition to *in situ* personalised medicine development.

## Conclusions

Reported is a novel method to synthesise and radiolabel MPN-based nanoparticles encapsulating a therapeutic for *in vivo* tracking using PET imaging, in a one-step reaction using an MIVM flow mixing approach. Characterisation showed that FRITH-MPNPs radiolabelled with gallium-68 both post-synthesis and *via* flow methods have comparable properties to unlabelled FRITH-MPNPs, with excellent radiochemical purity and serum stability, as well as enhanced radiolabelling yield when utilising the flow method. *In vivo* PET imaging and *ex vivo* analysis of a healthy murine model showed uptake in the liver, spleen and bone marrow as a result of MPS uptake, and an absence of renal excretion and bone uptake compared a free gallium-68 control confirming high radiochemical stability and purity *in vivo*. We propose that this efficient, one-step, flow, radiolabelling synthesis could be applied to *in situ* hospital treatment. With further development, this could unlock the ability to produce personalised theranostic agents directly in the clinic, with reduced need for highly trained chemists.

## Author contributions

RTMR and AS conceived and designed the study, secured funding, managed and supervised the project. ES contributed to the design of the study, drafted and revised the manuscript. ES, ACM and AM executed experiments and acquired data. ES conducted data analysis and interpretation. JS contributed to designing and fabricating the flow reactor. All authors read and approved the final manuscript prior to publication.

## Conflicts of interest

There are no conflicts of interest to declare.

## Supplementary Material

NR-018-D6NR01151A-s001

## Data Availability

The data supporting this article have been included as part of the supplementary information (SI). Supplementary information is available. See DOI: https://doi.org/10.1039/d6nr01151a.
