# Supplementary material for: One-step flow synthesis and [68Ga]Ga radiolabelling of metal phenolic network nanoparticles for in vivo PET tracking
Source: Nanoscale. 2026 Aug 27;18(37):20313–26. doi: 10.1039/d6nr01151a (PMC13549125; doi:10.1039/d6nr01151a)
Supplement: Supplementary file 1 [file NR-018-D6NR01151A-s001.pdf]

Supplementary Information (SI) for Nanoscale.
This journal is © The Royal Society of Chemistry 2026

Supporting Information

Characterising the degree of modification of HAd Capping Group

As shown in Fig. S1B, the ^{1}H-NMR spectrum displays a signal at approximately 2 ppm which relates to the methyl protons of the acetyl groups in HA, and the multiplet signals between 6.5-7.5 ppm correspond to the aromatic protons of the dopamine moiety (Fig. S1C & D). The ratio between peak areas shows a degree of modification of 27%. This was confirmed using a UV/Vis calibration curve of the dopamine absorbance at 280 nm (Fig. 1C) and calculated to be 26.48%, which agrees with the ^{1}H-NMR calculation, as well as being in line with literature values.

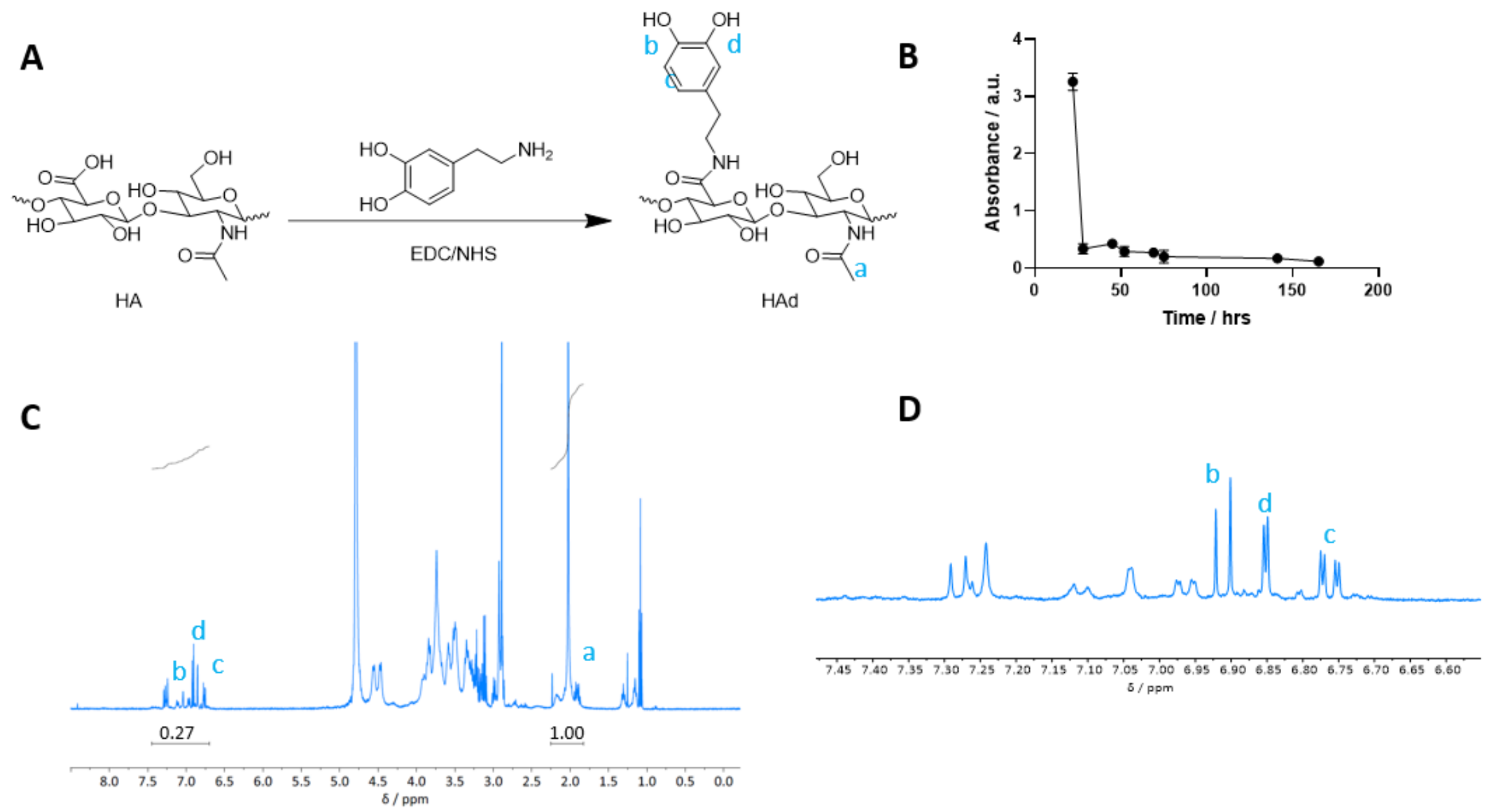

Figure S1: (A) Reaction scheme of the modification of hyaluronic acid (HA) with dopamine using EDC/NHS chemistry; **(B)** Graph showing how the absorbance of dopamine in the filtrate measured by UV/Vis at 280 nm changed with time before each water bath change; **(C)** 1H-NMR spectra of the lyophilised HAd, showing the integration of the peaks representing the aromatic groups and carbonyls of the HA, which is used to determine the degree of modification; **(D)** Aromatic region of the 1H-NMR shown in **(C).**

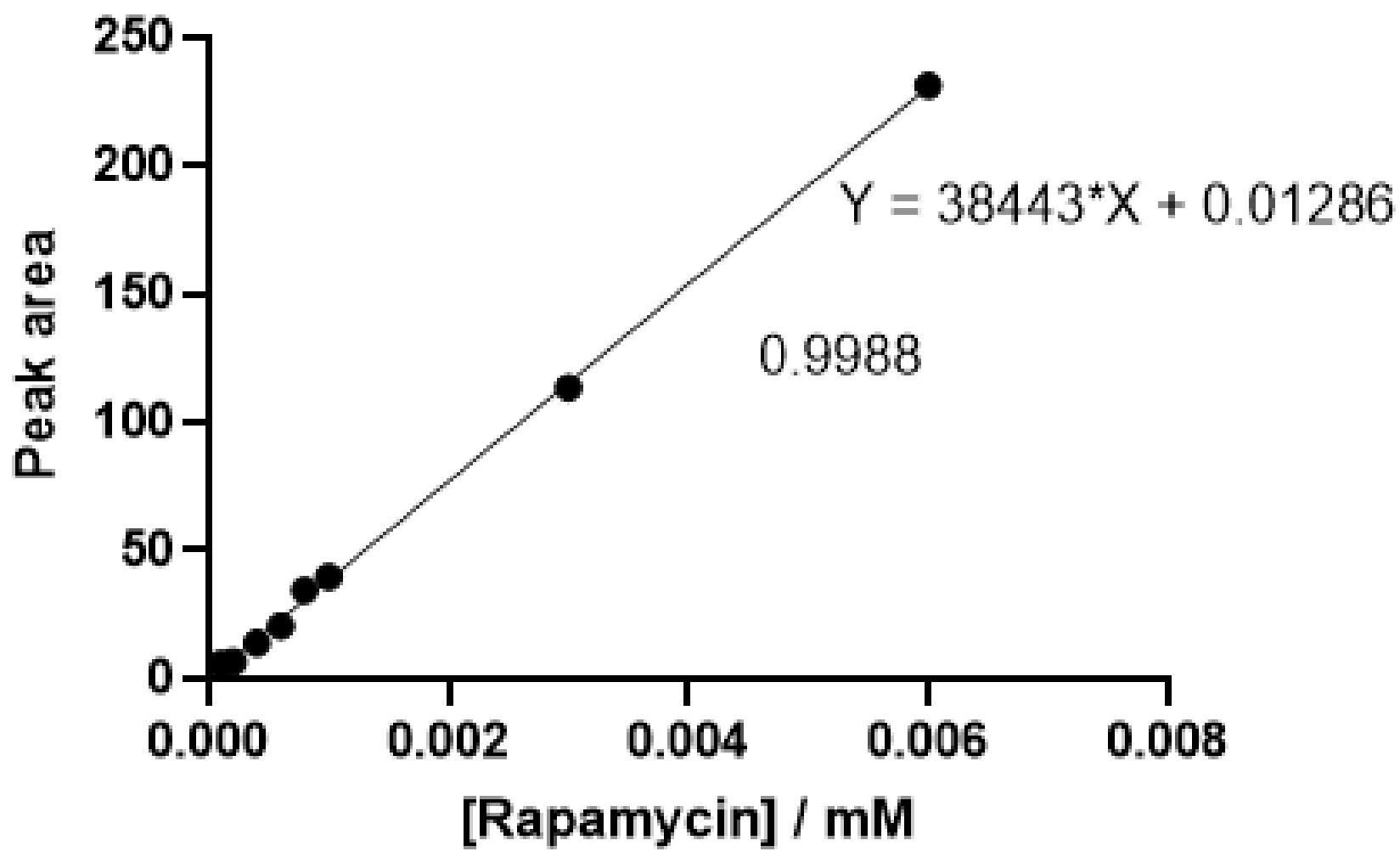

Figure S2: Calibration curve for the concentration of rapamycin across a range of calibrant concentrations ranging from the maximum rapamycin content in FRITH-MPNPs to the lowest detectable concentration of rapamycin using the HPLC method.

CAD designs for the MIVM flow mixer

Documented here is the full CAD designs for the MIVM flow mixer developed in this work for others to implement in their own work. Photographs are also provided for verification of the expected product.

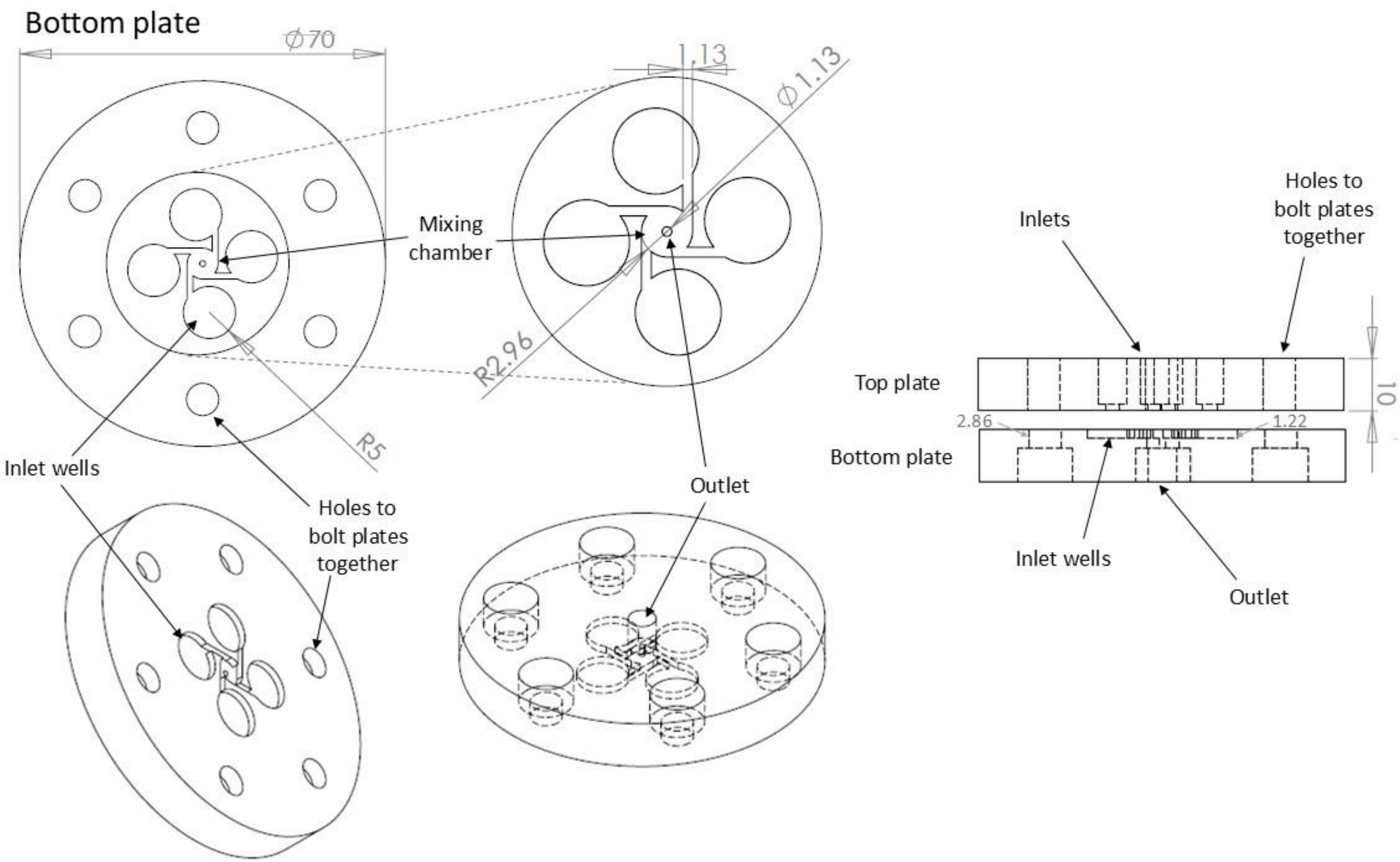
Bottom plate
Ø70
1.13
Ø1.13
R2.96
R5
Mixing chamber
Inlet wells
Holes to bolt plates together
Outlet
Inlets
Holes to bolt plates together
Top plate
Bottom plate
10
2.86
1.22
Inlet wells
Outlet

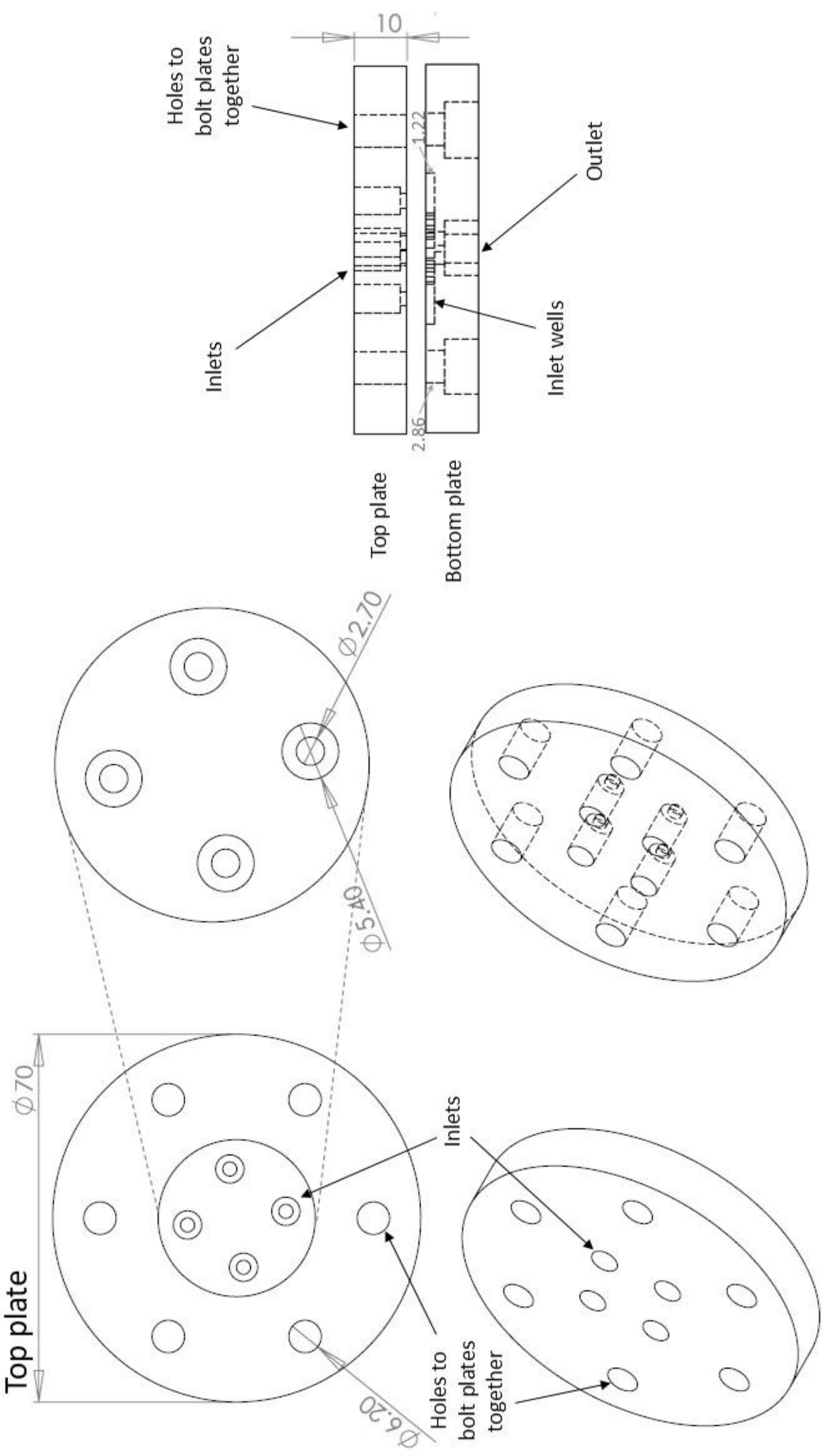
Top plate
ϕ70
Inlets
Holes to bolt plates together
ϕ6.20
ϕ5.40
ϕ2.70
Holes to bolt plates together
Inlets
10
1.22
2.86
Outlet
Inlet wells
Top plate
Bottom plate

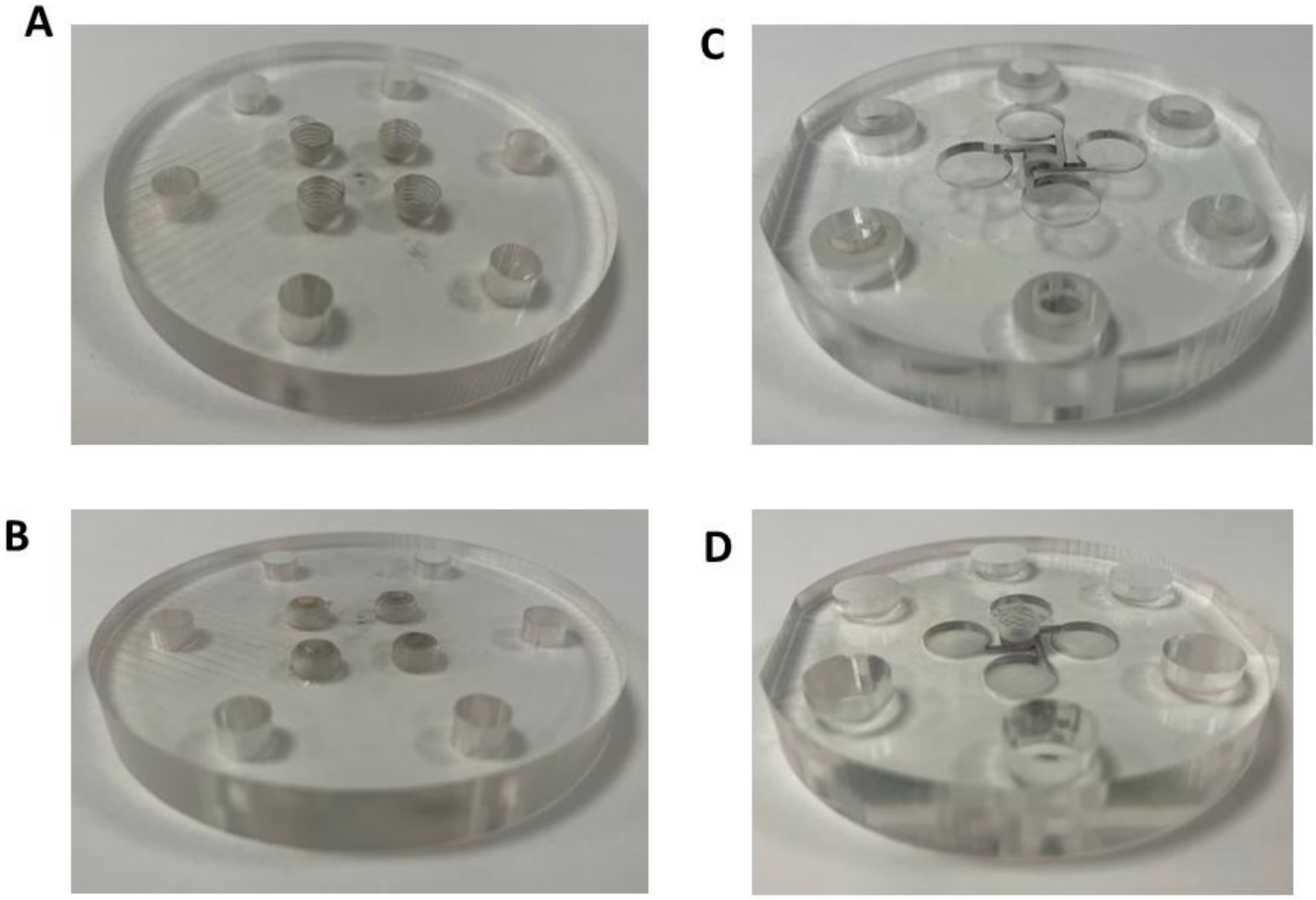

Figure S3: Photographs of the top and bottom plates of the MIVM **(A)** Top plate looking at top side; **(B)** Top plate looking at bottom side; **(C)** Bottom plate, looking at top side including wells and central mixing chamber; **(D)** Bottom plate looking at the bottom side, including the outlet port.

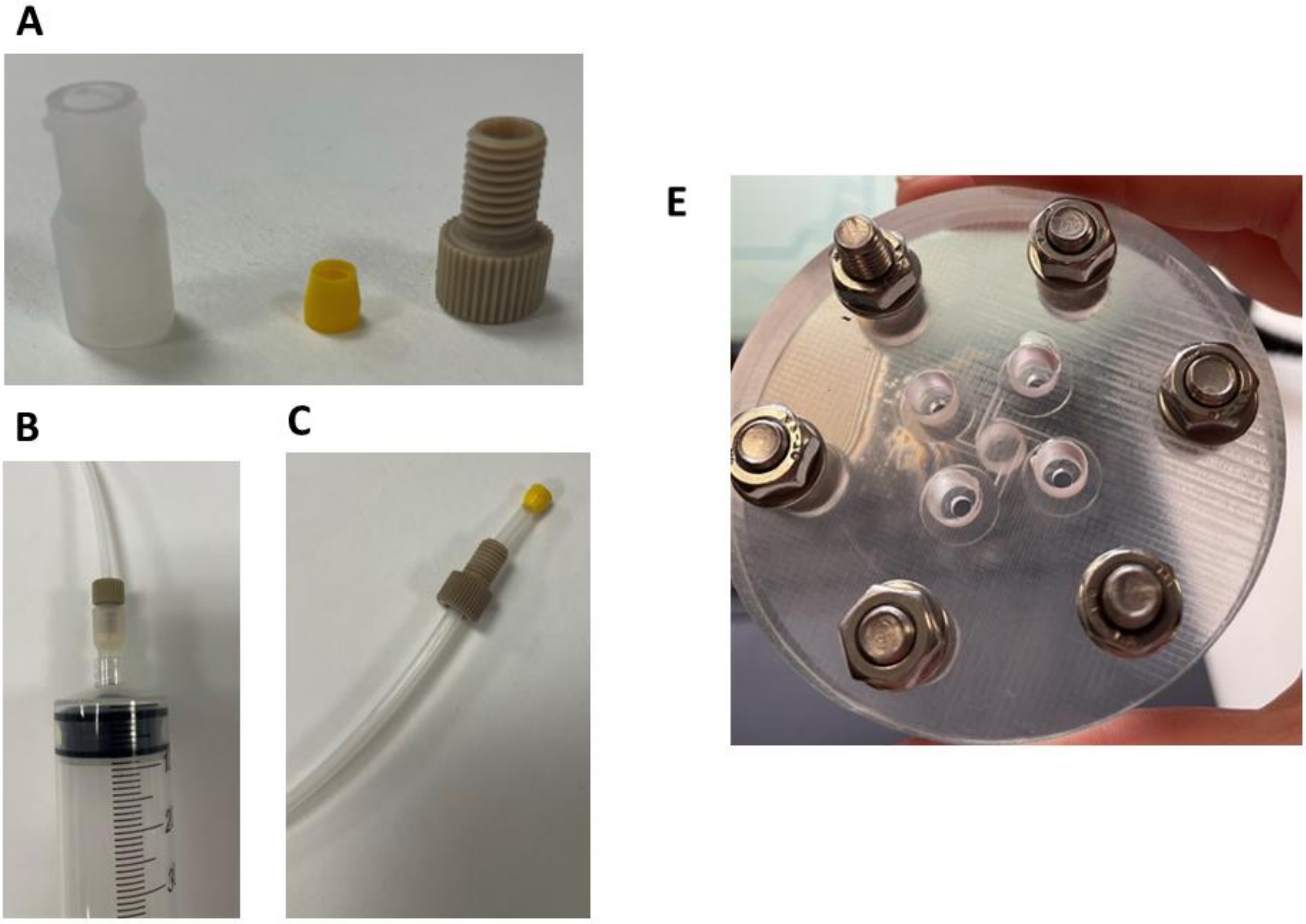

Figure S4: (A) Photograph of the components requried to connect the tubing to the syringes and mixer, including female luer to female adapter (left), ferrule (middle) and flangeless male nut (right); **(B)** Photograph of how the parts are assembled to connect the luer lock syringe to the tubing; **(C)** Photograph showing how the parts are assembled to connect the tubing to the mixer; **(D)** The plates after assembly using M6 sized bolts.

Details for safe shielding of [^{68}Ga]Ga-FRITH-MPNP *in situ* flow synthesis

Due to the high energy positron emission ofgallium-68, 5cm lead shielding is required to protect the user from receiving a high radiation dose. Due to the bulkiness and footprint of the syringe pump containing radioactivity, a specialised shielded workstation was created for this experiment. Below is the proposed design to prevent exposure and contamination, alongside a photograph of the actual set-up.

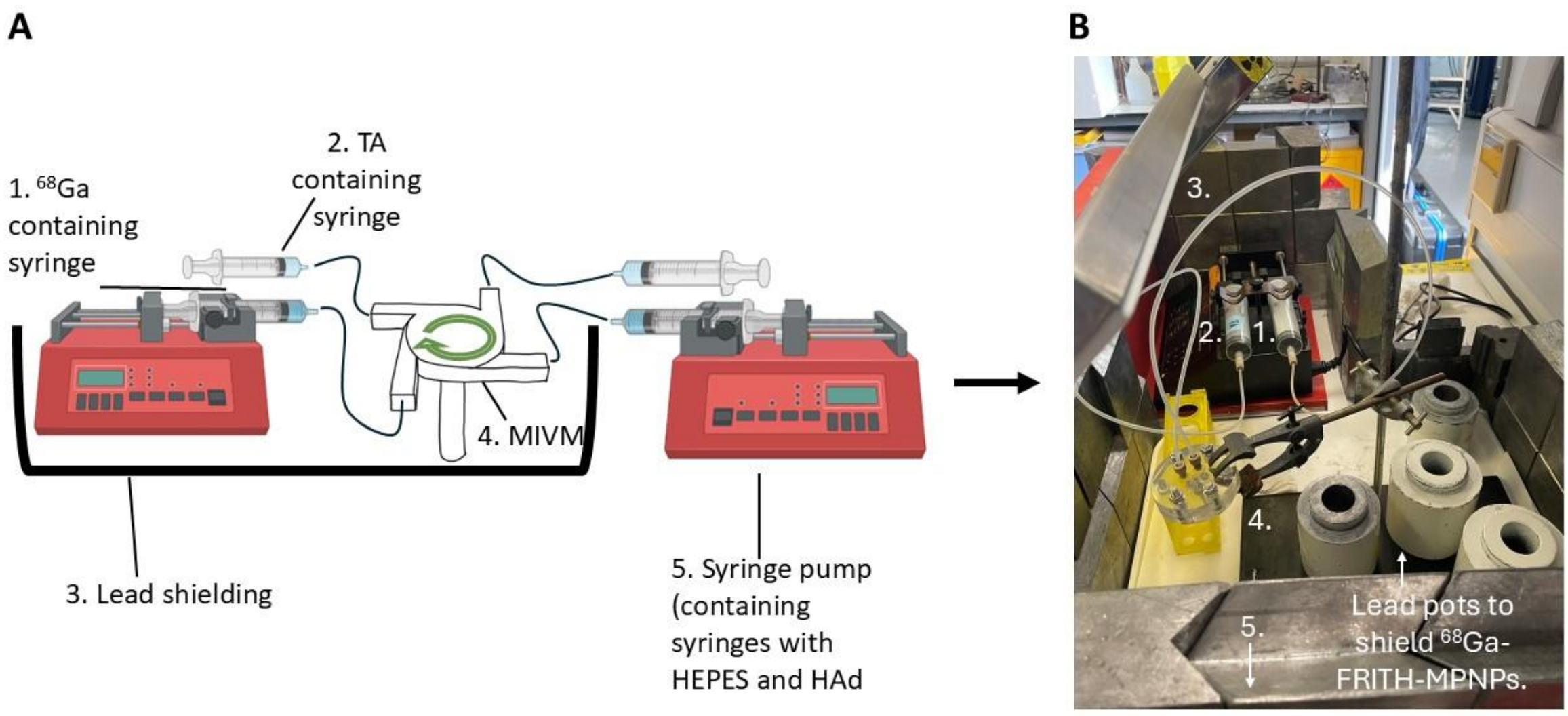

Figure S5: (A) Schematic of radiolabelling set-up for the flow mixer and syringe pumps; **(B)** Photograph of the actual flow mixing set-up.

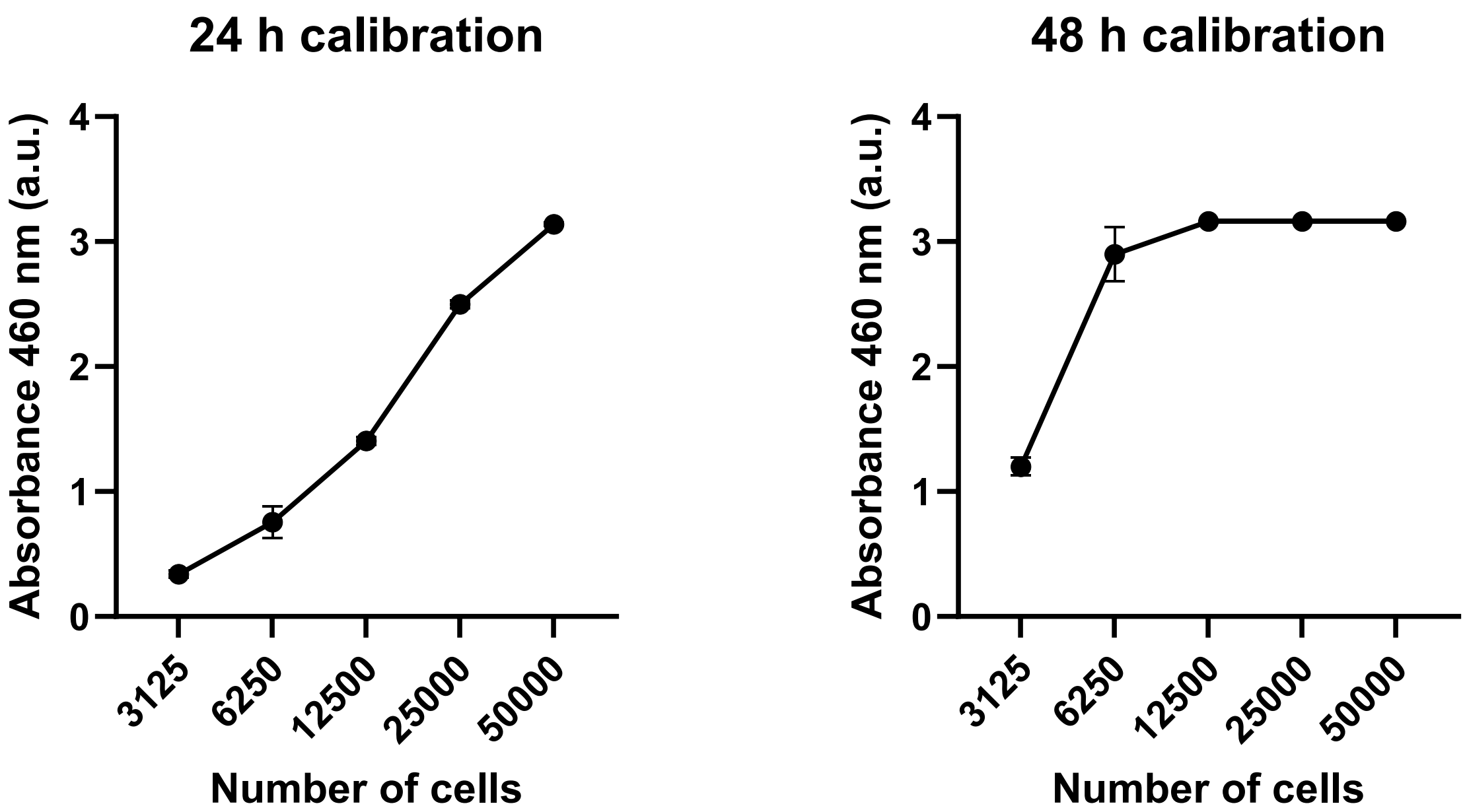

Figure S6: Calibration curves of J774a.1 cells seeded between 3000-50000 cells per well according to WST-8 cell viability assay at 24 and 48 h.

ROI data for processed images

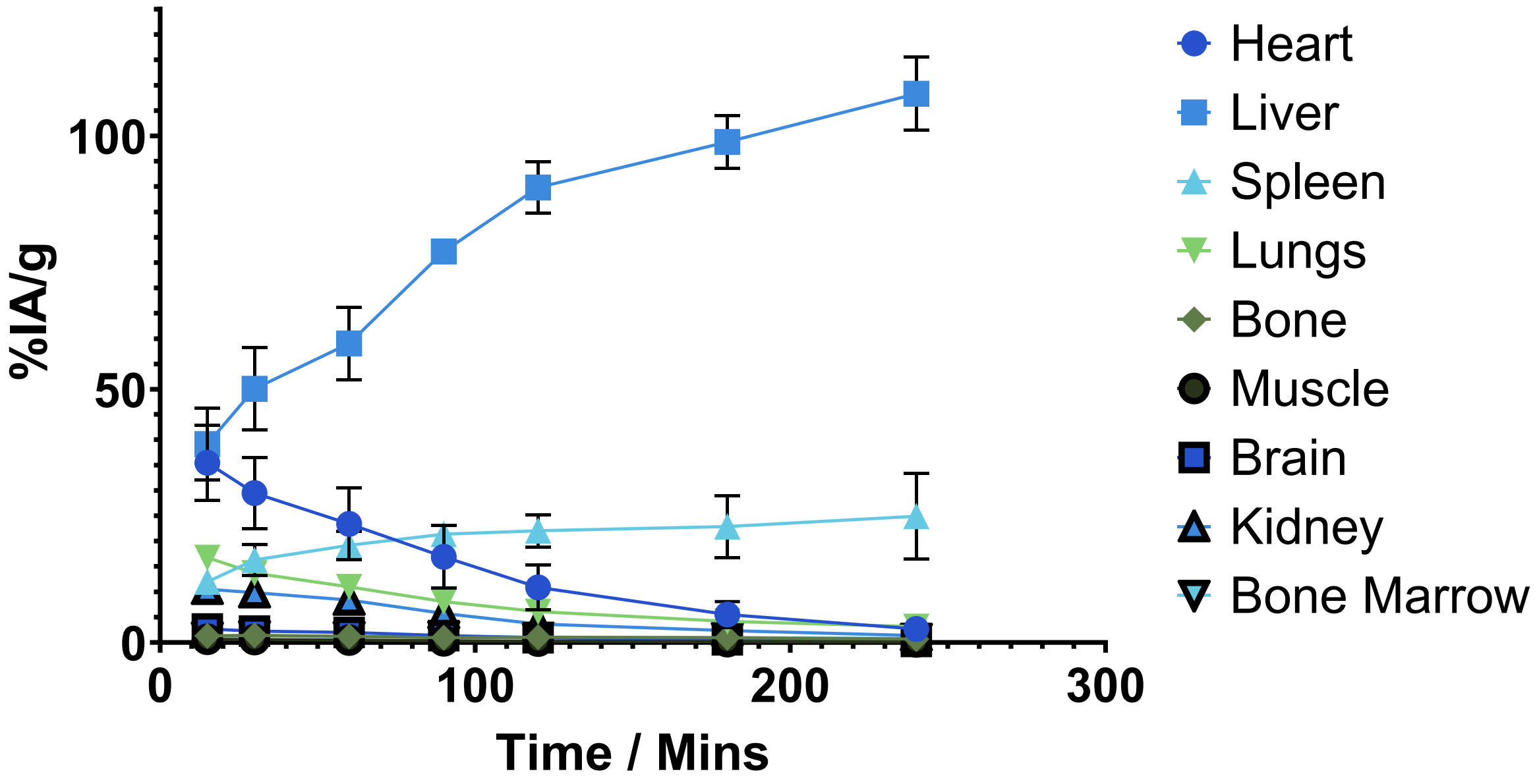

Figure S7: Percentage of injected activity per gram against time for each region of interest (ROI) determined from PET/CT image analysis.
